# TEMPO-derived spin labels linked to the nucleobases adenine and cytosine for probing local structural perturbations in DNA by EPR spectroscopy

**DOI:** 10.3762/bjoc.11.24

**Published:** 2015-02-09

**Authors:** Dnyaneshwar B Gophane, Snorri Th Sigurdsson

**Affiliations:** 1 University of Iceland, Department of Chemistry, Science Institute, Dunhaga 3, 107 Reykjavik, Iceland

**Keywords:** aminoxyl radical, ESR spectroscopy, nitroxide, nucleic acids, site-directed spin labeling (SDSL), spin labels, structure-dependent dynamics

## Abstract

Three 2´-deoxynucleosides containing semi-flexible spin labels, namely ^T^A, ^U^A and ^U^C, were prepared and incorporated into deoxyoligonucleotides using the phosphoramidite method. All three nucleosides contain 2,2,6,6-tetramethylpiperidine-1-oxyl (TEMPO) connected to the exocyclic amino group; ^T^A directly and ^U^A as well as ^U^C through a urea linkage. ^T^A and ^U^C showed a minor destabilization of a DNA duplex, as registered by a small decrease in the melting temperature, while ^U^A destabilized the duplex by more than 10 °C. Circular dichroism (CD) measurements indicated that all three labels were accommodated in B-DNA duplex. The mobility of the spin label ^T^A varied with different base-pairing partners in duplex DNA, with the ^T^A•T pair being the least mobile. Furthermore, ^T^A showed decreased mobility under acidic conditions for the sequences ^T^A•C and ^T^A•G, to the extent that the EPR spectrum of the latter became nearly superimposable to that of ^T^A•T. The reduced mobility of the ^T^A•C and ^T^A•G mismatches at pH 5 is consistent with the formation of ^T^AH^+^•C and ^T^AH^+^•G, in which protonation of N1 of A allows the formation of an additional hydrogen bond to N3 of C and N7 of G, respectively, with G in a syn-conformation. The urea-based spin labels ^U^A and ^U^C were more mobile than ^T^A, but still showed a minor variation in their EPR spectra when paired with A, G, C or T in a DNA duplex. ^U^A and ^U^C had similar mobility order for the different base pairs, with the lowest mobility when paired with C and the highest when paired with T.

## Introduction

The knowledge about structures and conformational dynamics of nucleic acids, as well as other biomolecules, is essential to understand their biological functions, including interactions with other molecules. The exact atom-to-atom structural information can be obtained by X-ray crystallography [1–6] and nuclear magnetic resonance (NMR) spectroscopy [7–12]. Electron paramagnetic resonance (EPR) and fluorescence spectroscopies are nowadays routinely used to study global structures and conformational changes under biologically relevant conditions through the determination of intermediate to long-range distances [13–30]. EPR spectroscopy can also give information about the relative orientation of two rigid spin labels [31–35]. Small angle X-ray scattering is also frequently used to study global structures of large molecules and molecular assemblies [36–39].

Local structural perturbations in nucleic acids can be studied with some of the aforementioned techniques. For example, NMR has been used to study hydrogen-bonding interactions [11,40–42], non-native base-pairing properties of nucleobases [43–46] and their dynamics [42,47–48]. Fluorescence spectroscopy, using environmentally sensitive fluorescent nucleosides has been used for detection of local structural perturbations [49–57], including the investigation of single-base mismatches [51,54,56,58–59], abasic sites [60] as well as nick sites in duplex DNA [61], and ligand-induced folding of riboswitches [62–63].

**Figure 1:**
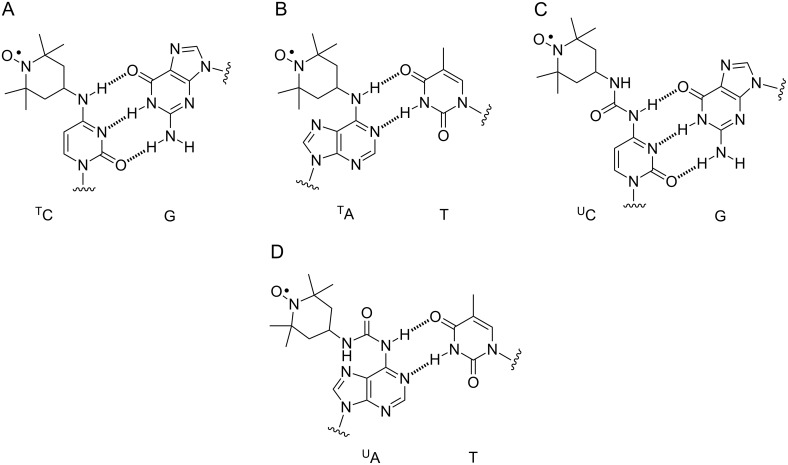
Base pairing of ^T^C with G (A), ^T^A with T (B), ^U^C with G (C) and ^U^A with T (D).

Continuous wave (CW) EPR spectroscopy can be used for the determination of structure-dependent dynamics based on the line-shape analysis of the EPR spectra [64–73]. The spin labels for such experiments are attached to the nucleotide via a flexible or a semi-flexible tether, which allows some motion of the spin label independent of the nucleic acid. Spin-label motion is affected by the local surroundings of the label, which in turn is reflected in the shape of the EPR spectra. We have previously used the spin label ^T^C [69], containing a 2,2,6,6-tetramethylpiperidine-1-oxyl (TEMPO) moiety conjugated to the exocyclic amino group of C (Figure 1A), to identify the base to which it is paired with in duplex DNA [69]. In other words, this label could not only distinguish between pairing with guanine and a mismatch but was also able to pinpoint the base-pairing partner. Furthermore, ^T^C revealed a flanking-base dependent variation in the EPR spectra, showing that minor structural variations in the local surroundings of a nucleic acid groove can be detected with spin labels by EPR spectroscopy.

Here, we describe the use of an analogous derivative of A, namely ^T^A, in which a TEMPO moiety is conjugated to the exocyclic amino group of 2´-deoxyadenosine (Figure 1B), to study local perturbations for a purine base in DNA. We show that ^T^A can indeed be used to differentiate between different base-pairing partners, albeit not as clearly as ^T^C. Lower pH causes noticeable changes in the EPR spectra for ^T^A, in particular for the ^T^A•G and ^T^A•C mismatches, presumably because of protonation of the base. We have also prepared urea-linked spin-labeled derivatives of 2´-deoxycytidine (^U^C) and 2´-deoxyadenosine (^U^A) and incorporated them into DNA duplexes (Figure 1C and D). These labels provide additional possibilities for hydrogen-bonding through the urea linkage but are also more flexible than ^T^A or ^T^C. In spite of the increased flexibility of ^U^C and ^U^A, inspection of the line-shape of their CW EPR spectra reveals subtle differences between the four base-pairing partners A, T, G and C when placed in a DNA duplex.

## Results and Discussion

### Synthesis of ^T^A, ^U^A, ^U^C and their corresponding phosphoramidites

**Scheme 1:**
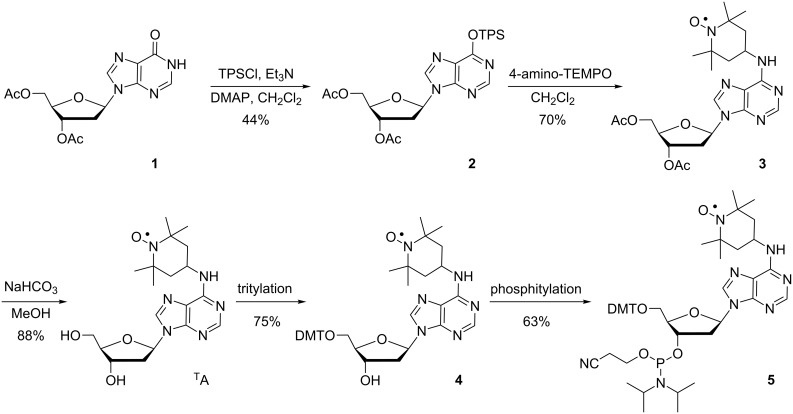
Synthesis of nucleoside ^T^A and its corresponding phosphoramidite **5**. TPS = 2,4,6-triisopropylbenzenesulfonyl.

The spin-labeled nucleoside ^T^A and its corresponding phosphoramidite were prepared by a previously reported procedure [74] with minor modifications. The synthesis started with the reaction between 3′,5′-diacetyl-2′-deoxyinosine (**1**) and 2,4,6-triisopropylbenzenesulfonyl chloride to obtain compound **2** (Scheme 1). Coupling of **2** with 4-amino-TEMPO gave **3** in good yields and deprotection of the acetyl groups gave ^T^A, which was tritylated and phosphitylated to yield compounds **4** and **5**, respectively.

**Scheme 2:**
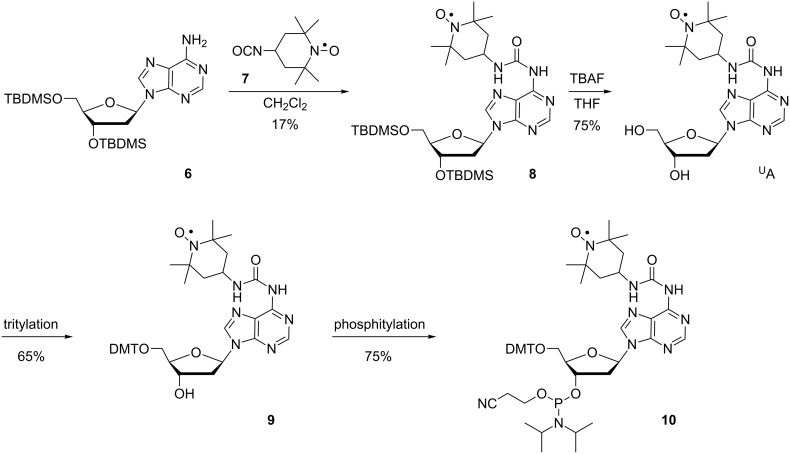
Synthesis of nucleoside ^U^A and its corresponding phosphoramidite **10**.

For the synthesis of ^U^A, 3′,5′-TBDMS-protected 2′-deoxyadenosine [75] (**6**) was reacted with 4-isocyanato-TEMPO (**7**) [64,76], which gave compound **8** in low yields (Scheme 2). Cleavage of the TBDMS groups using TBAF gave spin-labeled nucleoside ^U^A, which was tritylated to give compound **9** and phosphitylated to give phosphoramidite **10**.

**Scheme 3:**
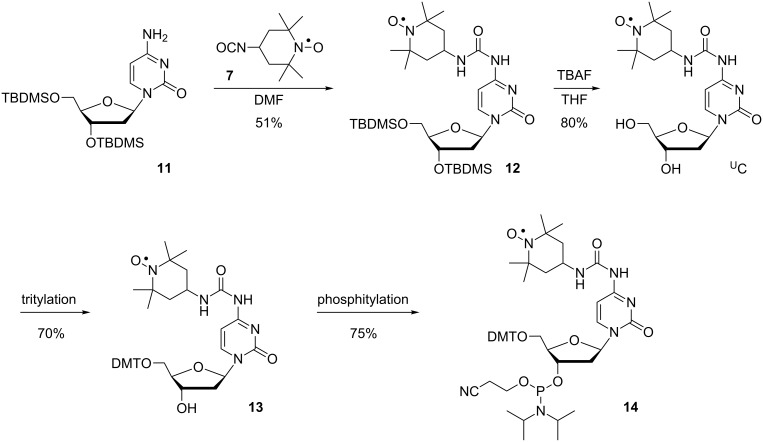
Synthesis of nucleoside ^U^C and its corresponding phosphoramidite **14**.

The synthesis of ^U^C, the urea-cytidine analogue, started by reaction of 3′,5′-TBDMS-protected 2′-deoxycytidine (**11**) with 4-isocyanato-TEMPO (**7**) [64,76] to give **12** in good yields (Scheme 3). Removal of the TBDMS groups using TBAF gave spin-labeled nucleoside ^U^C, which was tritylated to give compound **13** and subsequent phosphitylation yielded phosphoramidite **14**.

### Synthesis and characterization of ^T^A-, ^U^A- and ^U^C-containing oligonucleotides

The phosphoramidites of ^T^A (**5**), ^U^A (**10**) and ^U^C (**14**) were used to incorporate the spin-labeled nucleosides into DNA oligonucleotides using solid-phase synthesis [77]. The low stability of the nitroxide functional group in the TEMPO moiety towards acids lead to almost ca. 50% reduction of the nitroxide during oligonucleotide synthesis, which utilized dichloroacetic acid for the removal of the trityl groups. However, in spite of low yields, the spin-labeled oligonucleotides were readily separated from those containing the reduced spin label by denaturing polyacrylamide gel electrophoresis. The incorporation of ^T^A, ^U^A and ^U^C into DNA was confirmed by MALDI–TOF mass spectrometry (Table S1, Supporting Information File 1). Circular dichroism measurements showed that the incorporation of ^T^A, ^U^A and ^U^C does not alter the B-DNA conformation of DNA duplexes containing these modifications (Figure S1, Supporting Information File 1).

The thermal denaturation studies indicated only a minor destabilization of DNA the duplexes when ^T^A was paired with T (*T*_m_ was only 2.7 °C lower), compared to the natural nucleoside A (Table S2, Supporting Information File 1). The least stable base pairing was observed with the ^T^A•A mismatch, which showed destabilization by 8.2 °C, compared to an A•A mismatch. The duplex stability order as a function of base-paring with ^T^A was ^T^A•T > ^T^A•G > ^T^A•C > ^T^A•A, consistent with the order of stability previously reported for A [48]. Replacing C with ^U^C opposite G in a DNA duplex resulted in a 3.9 °C decrease in the melting temperature (*T*_m_) and a stability order of ^U^C•G > ^U^C•A > ^U^C•T > ^U^C•C (Table S3, Supporting Information File 1). In contrast to ^T^A and ^U^C, ^U^A had a large destabilizing effect on DNA duplexes (ca. 11 °C, Table S4, Supporting Information File 1). Interestingly, the melting temperatures of the DNA duplexes containing the base pairs ^U^A•T, ^U^A•G and ^U^A•A were nearly identical, whereas the ^U^A•C mismatch showed a further decrease in melting temperature of ca. 9 °C.

### EPR analysis of ^T^A-, ^U^C-and ^U^A-labeled DNA duplexes

**Figure 2:**
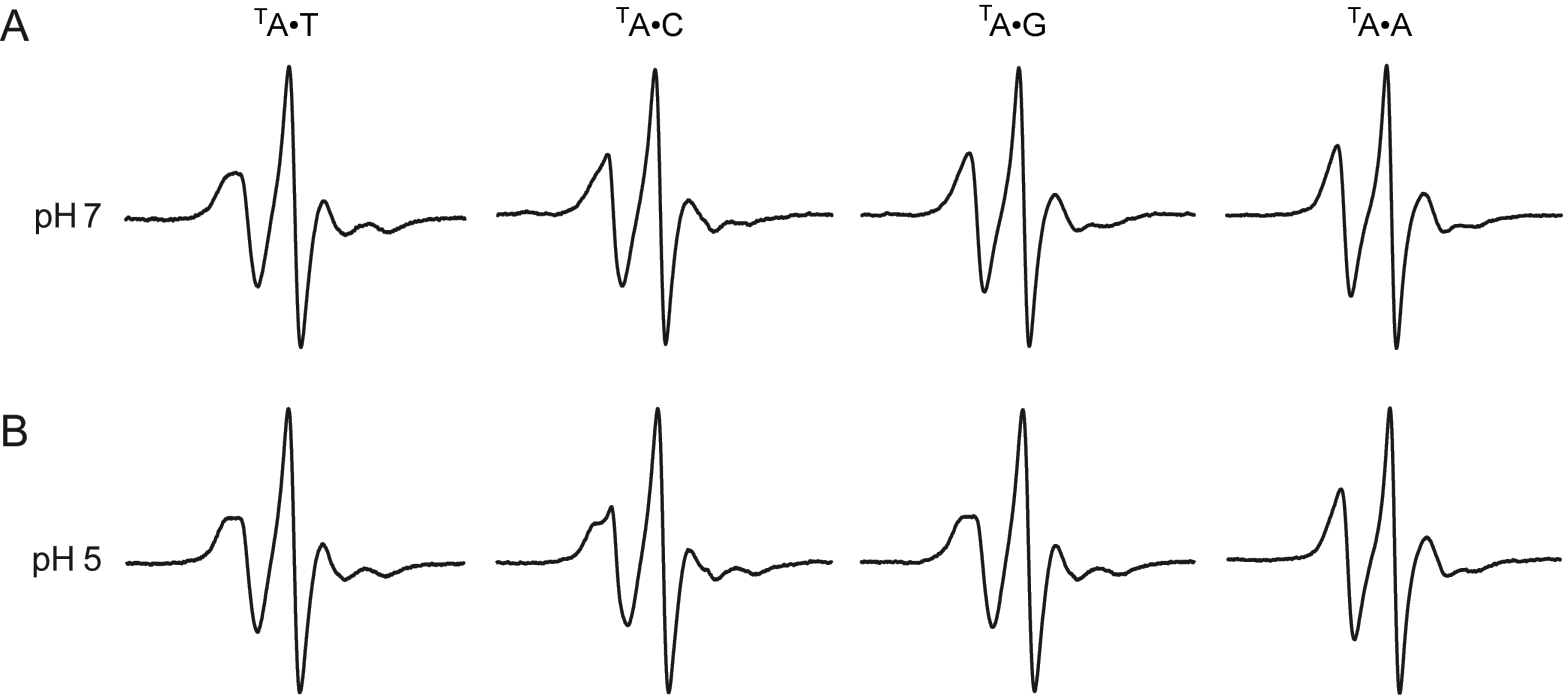
EPR spectra of 14-mer DNA duplexes 5′-d(GACCTCG^T^AATCGTG)•5′-d(CACGATYCGAGGTC), (10 mM phosphate, 100 mM NaCl, 0.1 mM Na_2_EDTA, pH 7.0 (A) and 5.0 (B)) at 10 °C, where ^T^A is paired with either Y = T, C, G or A.

To investigate the mobility of ^T^A in duplex DNA, we analyzed the EPR spectra of the four 14-mer DNA duplexes 5′-d(GACCTCG^T^AATCGTG)•5′-d(CACGATYCGAGGTC), where Y is either T, C, G or A (Figure 2). The EPR spectrum of the ^T^A•T pair was broadest, which is consistent with ^T^A forming a Watson–Crick base pair with T, and thereby restricting the rotation around the C6–N6 bond through hydrogen bonding to N6, and consequently slowing the motion of the label (Figure 1B). On the other hand, the spectrum of ^T^A•A was the narrowest and thereby indicating the highest mobility, while the EPR spectrum of ^T^A•G was slightly broader than ^T^A•A. Although base pairing schemes can be drawn for ^T^A•G and ^T^A•A that involve hydrogen bonding to N6 of ^T^A (Figure S2C and S2D, Supporting Information File 1) [44,48,78–80], the increased mobility could be the result of the label being pushed further into the major groove of the DNA duplex, due to the space-demanding purine–purine pairs [81], where the spin-label mobility would be less affected by the local surroundings.

**Figure 3:**
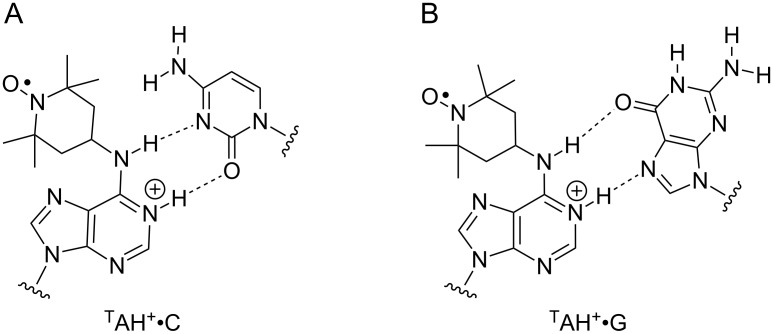
Possible base pairing of ^T^AH**^+^** with C (A) and G (B) at pH 5.

The mobility of ^T^A•C at pH 7, as judged by its EPR spectrum (Figure 2A), was between that of ^T^A•T and ^T^A•G. Previous NMR studies of the ^T^A•C mismatch at pH 8.5 [47] and 8.9 [82] showed one hydrogen bond, located between N6 of ^T^A and N3 of cytidine (Figure S2B, Supporting Information File 1). If ^T^A is protonated on N1 to form ^T^AH^+^, it could form a wobble-pair with C [47,83] (Figure 3A), which would be expected to decrease the mobility of the spin label. The apparent p*K*_a_ of the proton on N1 has been determined by NMR studies to be 7.2 [47], which means that more than half of the ^T^A•C pairs would be protonated at pH 7. To explore if further reduction in mobility (due to conversion of the ^T^A•C pair to the ^T^AH^+^•C pair) would be detected by EPR, its spectrum was also recorded at pH 5 (Figure 2B). Indeed, comparison of the EPR spectra of ^T^A•C at pH 5 and pH 7 clearly shows further broadening at the lower pH, almost to that of the ^T^A•T pair, and is consistent with the formation of the ^T^AH^+^•C pair.

The ^T^A•T and ^T^A•A pairs had similar EPR spectra at pH 7 and 5 (Figure 2B). However, significant broadening was observed in the EPR spectrum of the ^T^A•G mismatch at pH 5. In fact, the spectrum of ^T^A•G is nearly superimposable to that of the ^T^A•T pair, indicating reduction in mobility due to formation of another hydrogen bond at pH 5. Studies by NMR and X-ray crystallography have shown that ^T^AH^+^•G pairs form when ^T^A•G mismatches are incubated at pH below 5.6, in which the G has flipped into a syn conformation and the O6 and N*7* form Hoogsteen hydrogen bonds (Figure 3B) [43–44,78]. Thus, the formation of the ^T^AH^+^•G pair can be readily detected by EPR spectroscopy.

**Figure 4:**
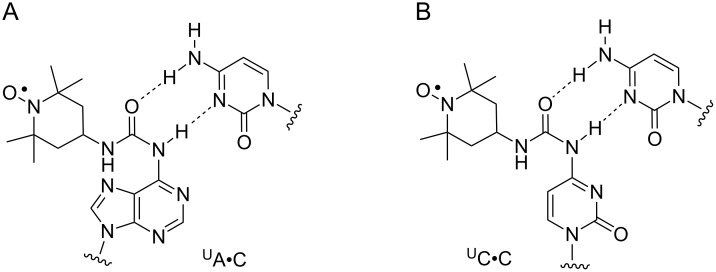
Possible base pairing of ^U^A and ^U^C with C.

Nucleosides ^U^A and ^U^C, the new and readily prepared spin labels that are described here, contain a stable urea linkage that provides additional hydrogen-bonding possibilities. In particular, pairing of ^U^A and ^U^C to C, which has been shown by NMR to form a hydrogen bond between its N3 and the proton on N6 of A [83] as well as N4 of C [69,84], should enable the oxygen in the urea moiety of both ^U^A and ^U^C to pair with a N4 proton of C (Figure 4A and B). Such hydrogen bonding should have an effect on the spin label mobility and be manifested in the line width of the EPR spectra.

**Figure 5:**
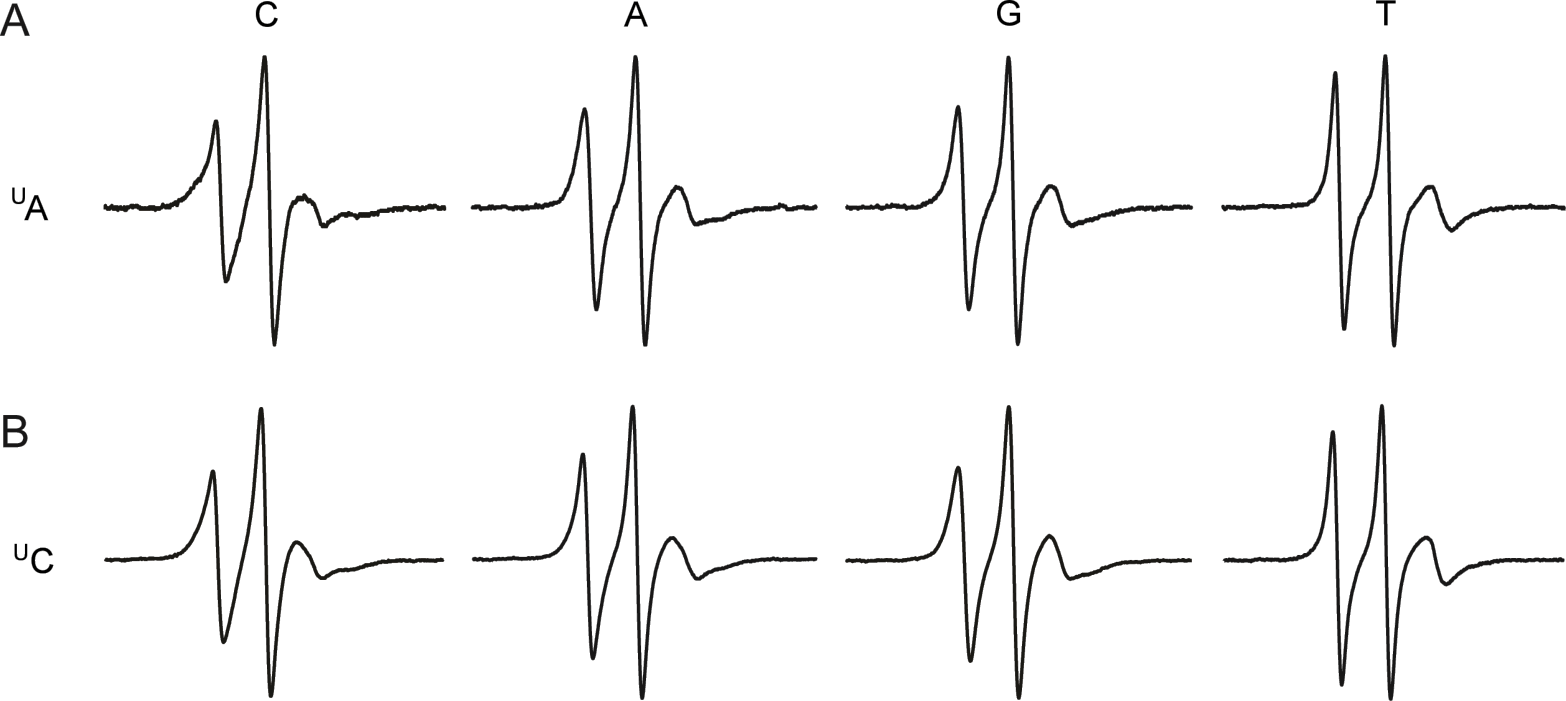
EPR spectra of 14-mer DNA duplex 5′-d(GACCTCG^U^AATCGTG)•5′-d(CACGATYCGAGGTC) (A), and 5′-d(GACCTCG^U^CATCGTG)•5′-d(CACGATYCGAGGTC) (B), (10 mM phosphate, 100 mM NaCl, 0.1 mM Na_2_EDTA, pH 7.0) at 10 °C, where ^U^A or ^U^C is paired with either Y = T, C, G or A .

Therefore, we recorded the EPR spectra of duplexes 5′-d(GACCTCGXATCGTG)•5′-d(CACGATYCGAGGTC), where X is either ^U^A or ^U^C, and Y is either C, A, G or T (Figure 5). Indeed, the lowest mobility, i.e., the widest spectra was observed for both ^U^A•C and ^U^C•C, providing circumstantial evidence for hydrogen bonding of C to the urea linkage. Interestingly, the same mobility order was observed for both ^U^A and ^U^C: X•C < X•A ≤ X•G < X•T. Pairing with T resulted in a high mobility for both ^U^A and ^U^C, whereas pairing with either A or G, caused mobility intermediate between that of C and T. As expected, the EPR spectra of the duplexes containing the urea-linked spin labels (^U^A and ^U^C) were narrower than for the N6-TEMPO-dA (^T^A) labeled duplexes. The extended urea linker not only contains more rotatable single bonds but also projects the spin label further out of the major groove, where it is less constrained sterically by the DNA.

## Conclusion

Three spin-labeled deoxynucleosides, ^T^A, ^U^A and ^U^C, were prepared and incorporated into oligonucleotides by the phosphoramidite method. While ^U^A resulted in a major decrease in the melting temperature of a DNA duplex when incorporated opposite to C, CD measurements revealed that all three spin labels were accommodated in a B-form DNA duplex. The mobility of the spin label ^T^A was highly base-pair sensitive, allowing detection of its respective base-pairing partner in duplex DNA. Moreover, the mobility of ^T^A was significantly reduced when paired with C or G in a DNA duplex at pH 5. This finding is consistent with protonation of ^T^A and subsequent participation of the proton in hydrogen-bonding with C and G. The urea-linked ^U^A and ^U^C spin labels showed a similar mobility order when paired with A, G, C or T in a DNA duplex, with the lowest mobility when paired with C and the highest for T. These results show that the three labels, in particular ^T^A, can report minor changes in their microenvironment, such as protonation, when placed in structured regions of nucleic acids.

## Supporting Information

File 1 Experimental part.
