# Supplementary material for: TEMPO-derived spin labels linked to the nucleobases adenine and cytosine for probing local structural perturbations in DNA by EPR spectroscopy
Source: Beilstein J Org Chem. 2015 Feb 9;11:219–27. doi: 10.3762/bjoc.11.24 (PMC4362019; doi:10.3762/bjoc.11.24)
Supplement: File 1 — Experimental part. [file Beilstein_J_Org_Chem-11-219-s001.pdf]

Supporting Information

for

TEMPO-derived spin labels linked to the nucleobases adenine and cytosine for probing local structural perturbations in DNA by EPR spectroscopy

Dnyaneshwar B. Gophane and Snorri Th. Sigurdsson*

Address: University of Iceland, Department of Chemistry, Science Institute, Dunhaga 3, 107 Reykjavik, Iceland

Email: Snorri Th. Sigurdsson - snorrisi@hi.is

*Corresponding author

Experimental part

Contents

List of abbreviations..

General materials and methods .... page S4

Synthesis and purification of oligonucleotides .... page S5

MALDI-TOF MS analysis of oligonucleotides .... page S5

CD measurements .... page S6

T_m measurements .... page S7

Synthetic procedures .... page S9

Compound **1**.. page S9

Compound **2**.. page S11

Compound **3**.. page S13

Spin-labeled nucleoside $^{\mathbf{T}}$**A**. .... page S15

DMT- ^{T}A (**4**). .... page S17

^{T}A phosphoramidite (**5**) .... page S19

Compound **6**.. page S21

Compound **8**.. page S23

Spin-labeled nucleoside ^{U}A .... page S25

DMT- ^{U}A (**9**).. page S27

^{U}A phosphoramidite (**10**) .... page S29

Compound **11**.. page S31

Compound **12**.. page S33

Spin-labeled nucleoside ^{U}C... page S35

DMT-^{U}C (**13**) .... page S37

^{U}C phosphoramidite (**14**) .... page S39

References .... page S41

List of abbreviations

CD	circular dichroism
CW	continuous-wave
DMSO	dimethylsulfoxide
DMTrCl	4,4′-dimethoxytrityl chloride
DMAP	4-dimethylaminopyridine
EPR	electron paramagnetic resonance
HRMS-ESI	high resolution electrospray ionization mass spectrometry
MALDF–TOF	matrix-assisted laser desorption/ionization - time of flight
NMR	nuclear magnetic resonance
t-BuOOH	*tert*-butylhydroperoxide
TBAF	tetrabutylammonium fluoride
TBDMS	*tert*-butyldimethyl silyl
^{T}A	TEMPO-linked A
^{U}A	urea-TEMPO-linked A
^{U}C	urea-TEMPO-linked C

General materials and methods

All chemicals were purchased from Sigma-Aldrich, Acros or Fluka and were used without further purification. Water was purified on an EASYpure RoDi Water Purification Systems. Thin-layer chromatography (TLC) was carried out using glass plates pre-coated with silica gel (0.25 mm, F-254) from Silicycle. Compounds were visualized by UV light and staining with *p*-anisaldehyde. Flash column chromatography was performed using ultra pure flash silica gel (Silicycle, 230–400 mesh size, 60 Å). Dichloromethane and pyridine were freshly distilled over calcium hydride prior to use. Anhydrous triethylamine, *n*-hexane and ethyl acetate were used directly as received. All moisture and air sensitive reactions were carried out in oven dried glassware under an inert argon atmosphere. Nuclear magnetic resonance (NMR) spectra were recorded on a Bruker Avance 400 MHz spectrometer. ^{1}H NMR chemical shifts are reported in parts per million (ppm) relative to the residual proton signal of solvents $CDCl_3$ (7.26 ppm), d_6-DMSO (2.50 ppm) or d_4-MeOH (3.31 and 4.84 ppm) for ^{1}H NMR and $CDCl_3$ (77.0 ppm), d_6-DMSO (39.43 ppm) or d_4-MeOH (49.05) for ^{13}C NMR. ^{31}P NMR chemical shifts are reported relative to 85% H_3PO_4 as an external standard. Commercial grade $CDCl_3$ was passed over basic alumina shortly before use with tritylated compounds. NMR spectra of compounds containing nitroxide radical show significant broadening, sometimes to the extent that some nuclei are not seen in the spectra [1-3]. Therefore, integration of ^{1}H NMR spectra of nitroxides is not reported. Mass spectrometric analyses of all organic compounds were performed on an HR-ESI-MS (Bruker, MicroTof-Q) in positive ion mode. UV–vis spectra were recorded on a PerkinElmer Lambda 25 UV–vis spectrometer. CD spectra were recorded on a JASCO J-810 spectropolarimeter at 25 °C with a path length of 1 mm (Hellma), 10 scans, scanned from 500 nm to 200 nm with response of 1s, data pitch of 0.1 nm and band width of 1.0 nm. Molecular weight (MW) of oligonucleotides were determined by MALDI–TOF analysis (Bruker, Autoflex III).

Synthesis and purification of oligonucleotides

MALDI–TOF MS analysis of oligonucleotides

The incorporation of ^{T}A, ^{U}A and ^{U}C into oligonucleotides was confirmed by MALDI–TOF MS analysis. The instrument was calibrated with an external standard prior to measurements. The calculated and observed monoisotopic masses of oligonucleotides are listed in Table S1.

Table S1. Monoisotopic masses of oligonucleotides

Sr. No.	Sequence	Monoisotopic mass (M+H) (calculated)	Monoisotopic mass (M+H) (observed)
1	5'-d(GACCTCG$^{\mathbf{T}}$**A**ATCGTG)	4418.89	4418.88
2	5'-d(GACCTCG$^{\mathbf{T}}$**A**CTCGTG)	4392.72	4392.76
3	5'-d(GACCTCT$^{\mathbf{T}}$**A**ATCGTG)	4391.73	4391.88
4	5'-d(GACCTCG$^{\mathbf{U}}$**A**ATCGTG)	4459.80	4460.65
5	5'-d(GACCTCG$^{\mathbf{U}}$**A**CTCGTG)	4435.87	4436.95
6	5'-d(GACCTCT$^{\mathbf{U}}$**A**ATCGTG)	4434.87	4435.78
7	5'-d(GACCTCG$^{\mathbf{U}}$**C**ATCGTG)	4436.00	4436.83
8	5'-d(GACCTCG$^{\mathbf{U}}$**C**CTCGTG)	4411.99	4411.40
9	5'-d(GACCTCT$^{\mathbf{U}}$**C**ATCGTG)	4410.99	4410.78

CD measurements

To determine if the ^{T}A, ^{U}A and ^{U}C spin labels had any effect on the DNA duplex conformation, circular dichroism (CD) spectra of a 14-mer unmodified and a spin-labeled DNA duplex were recorded. The DNAs samples (2.5 nmol of duplex) were dissolved in 100 µL of phosphate buffer (10 mM phosphate, 100 mM NaCl, 0.1 mM Na_2EDTA, pH 7.0) and annealed using the following annealing protocol: 90 °C for 2 min, 60 °C for 5 min, 50 °C for 5 min, 40 °C for 5 min, 22 °C for 15 min. The annealed samples were diluted to 200 µL with the same buffer. The spectra of ^{T}A, ^{U}A and ^{U}C modified DNAs and unmodified DNAs both possessed negative and positive molar ellipticities at ca. 250 and 280 nm respectively (Figure S1), characteristic of a right-handed B-DNA.

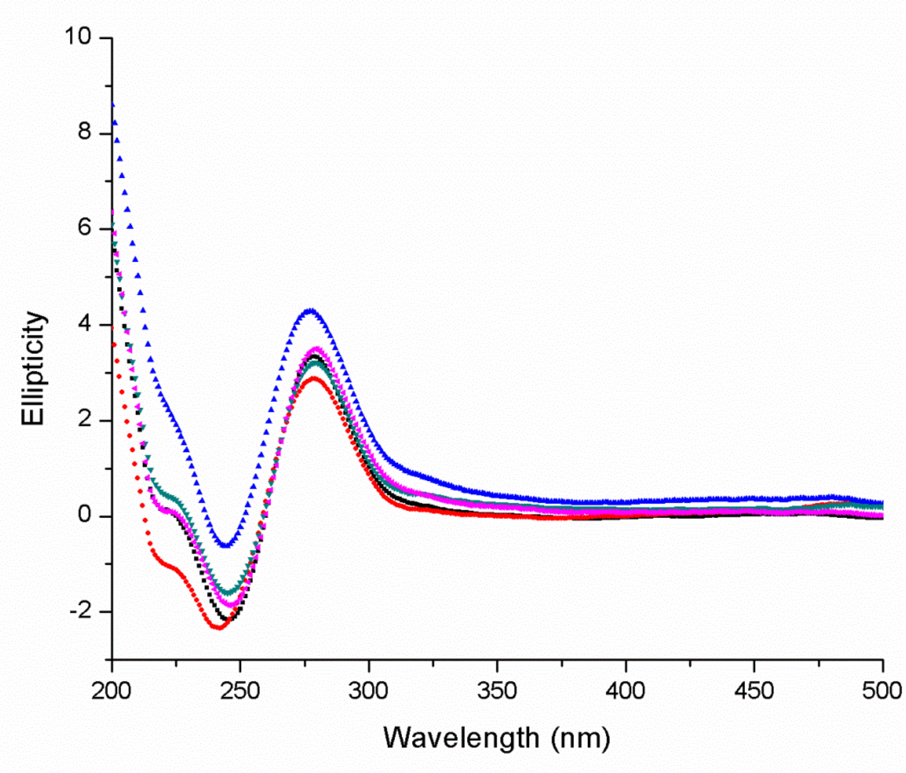

Figure S1. CD spectra of 14-mer DNA duplexes 5′-d(GACCTCG**X**ATCGTG)•5′-d(CACGAT**Y**CGAGGTC), where **X** = **A** (black), $^{\mathbf{T}}$**A** (red), $^{\mathbf{U}}$**A** (blue), **C** (green) and $^{\mathbf{U}}$**C** (pink) and **Y** = **T** or **G** accordingly.

T_m measurements

To determine if the ^{T}A, ^{U}A and ^{U}C affected the stability of DNA duplexes, the thermal denaturation curves of unmodified and spin-labeled oligomers were determined. DNA samples (2.0 nmol of each strand) were dissolved in phosphate buffer (100 µL) (10 mM phosphate, 100 mM NaCl, 0.1 mM Na_2EDTA, pH 7.0 or pH 5.0), annealed and diluted to 1.0 mL with the phosphate buffer (pH 7.0 or pH 5.0 accordingly) and degassed with argon. The samples were heated up from 20 °C to 90 °C (1.0 °C/min) and absorbance at 260 nm was recorded at 1.0 °C intervals. Table S2, S3 and S4 show melting temperatures (T_m) of ^{T}A, ^{U}C and ^{U}A spin-labeled and unmodified 14-mer DNA duplexes respectively.

Table S2. Melting temperatures of DNA duplexes containing $^{\mathbf{T}}$**A** instead of **A**

Duplex	Sequence	T_m (°C)[a] (unmodified) at pH 7	T_m(°C)[b] (modified)	ΔT_m (°C)	T_m (°C)[a] (unmodified) at pH 5
1	5′-d(GACCTCG**X**ATCGTG)<br>3′-d(CTGGAGCTTAGCAC)	55.7 ± 0.7	53.0 ± 1.0	− 2.7	NA
2	5′-d(GACCTCG**X**ATCGTG)<br>3′-d(CTGGAGCGTAGCAC)	50.0 ± 1.0	47.0 ± 1.0	− 3.0	52.0 ± 0.5
3	5′-d(GACCTCG**X**ATCGTG)<br>3′-d(CTGGAGCATAGCAC)	47.0 ± 1.0	38.8 ± 0.8	− 8.2	NA
4	5′-d(GACCTCG**X**ATCGTG)<br>3′-d(CTGGAGCCTAGCAC)	44.7 ± 0.3	40.7 ± 0.7	− 4.0	51.0 ± 0.5

[a]T_m and [b]T_m is the melting temperature of **A**-and $^{\mathbf{T}}$**A**-containing oligonucleotides, respectively, and ΔT_m is difference in T_m between unmodified and modified duplexes.

Table S3. Melting temperatures of DNA duplexes containing $^{\mathbf{U}}$**C** instead of **C**

Duplex	Sequence	T_m (°C)[a] (unmodified)	T_m (°C)[b] (unmodified)	ΔT_m (°C)
1	5′-d(GACCTCG**X**ATCGTG)<br>3′-d(CTGGAGCGTAGCAC)	61.7 ± 0.7	57.8 ± 0.7	− 3.9
2	5′-d(GACCTCG**X**ATCGTG)<br>3′-d(CTGGAGCTTAGCAC)	46.3 ± 0.7	43.8 ± 0.7	− 2.5
3	5′-d(GACCTCG**X**ATCGTG)<br>3′-d(CTGGAGCATAGCAC)	48.0 ± 0.0	45.3 ± 0.7	− 2.7
4	5′-d(GACCTCG**X**ATCGTG)<br>3′-d(CTGGAGCCTAGCAC)	44.0 ± 0.0	41.8 ± 0.7	− 2.2

[a]T_m and [b]T_M is the melting temperature of C-and ^{U}C-containing oligonucleotides, respectively, and ΔT_m is difference in T_m between unmodified and modified duplexes.

Table S4. Melting temperatures of DNA duplexes containing ^{U}**A** instead of **A**

Duplex	Sequence	T_m (°C)[a] (unmodified)	T_m (°C)[b] (modified)	ΔT_m (°C)
1	5′-d(GACCTCG**X**ATCGTG)<br>3′-d(CTGGAGCTTAGCAC)	55.7 ± 0.7	45.0 ± 0.0	− 10.7
2	5′-d(GACCTCG**X**ATCGTG)<br>3′-d(CTGGAGCGTAGCAC)	50.0 ± 1.0	45.0 ± 1.0	− 5.0
3	5′-d(GACCTCG**X**ATCGTG)<br>3′-d(CTGGAGCATAGCAC)	47.0 ± 1.0	45.3 ± 1.0	− 1.7
4	5′-d(GACCTCG**X**ATCGTG)<br>3′-d(CTGGAGCCTAGCAC)	44.7 ± 0.3	36.3 ± 0.7	− 8.4

[a]T_m and [b]T_m is the melting temperature of A-and ^{U}A-containing oligonucleotides, respectively, and ΔT_m is difference in T_m between unmodified and modified duplexes.

Base pairing of ^{T}A at pH 7

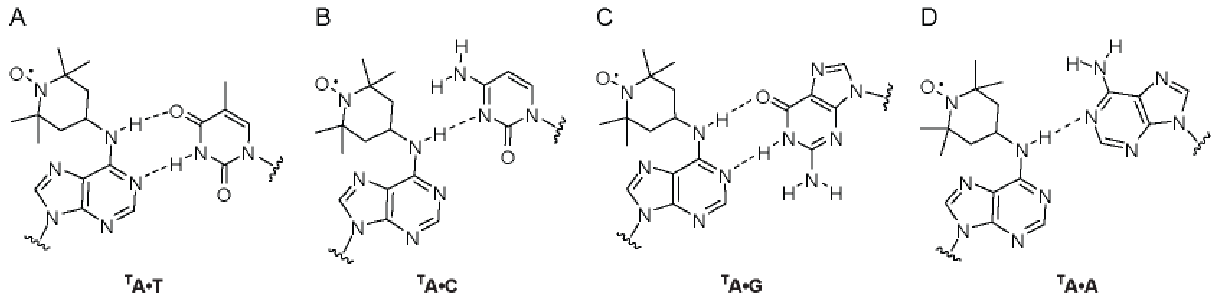

Figure S2. Possible base pairing of ^{T}A with T, C, G and A at pH 7.

Synthetic procedures

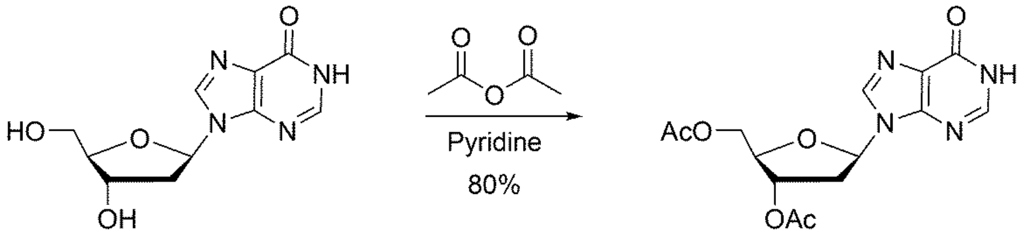

1

Compound 1. To a solution of inosine (1.0 g, 3.97 mmol) in dry pyridine (3.2 mL, 40.4 mmol) was added acetic anhydride (2.42 mL, 23.8 mmol) at 0 °C. The reaction mixture was stirred at 22 °C for 18 h and the solvent was removed *in vacuo*. The residue was purified by flash silica gel column chromatography using a gradient elution (CH_2Cl_2:MeOH; 98:2 to 90:10) to give compound **1** as a white solid (1.06 g, 80% yield).

^{1}H NMR (400 MHz, $CDCl_3$): δ 13.06 (s, 1H), 8.26 (s, 1H), 8.03 (s, 1H), 6.40 (dd, J = 7.8, 6.1 Hz, 1H), 5.41 (dd, J = 4.0, 2.2 Hz, 1H), 4.53 – 4.19 (m, 3H), 2.89 (ddd, J = 14.2, 7.8, 6.5 Hz, 1H), 2.64 (ddd, J = 14.1, 6.0, 2.6 Hz, 1H), 2.13 (s, 3H), 2.09 (s, 3H).

^{13}C NMR (101 MHz, $CDCl_3$): δ 170.64, 170.48, 159.26, 148.83, 145.57, 138.43, 125.42, 84.95, 82.92, 74.58, 63.89, 38.05, 21.11, 20.98.

HR-ESI-MS $(M + Na)^+$: calcd. for $C_{14}H_{16}N_4O_6Na$ 359.0962, found 359.0969.

^{1}H NMR spectrum of 1

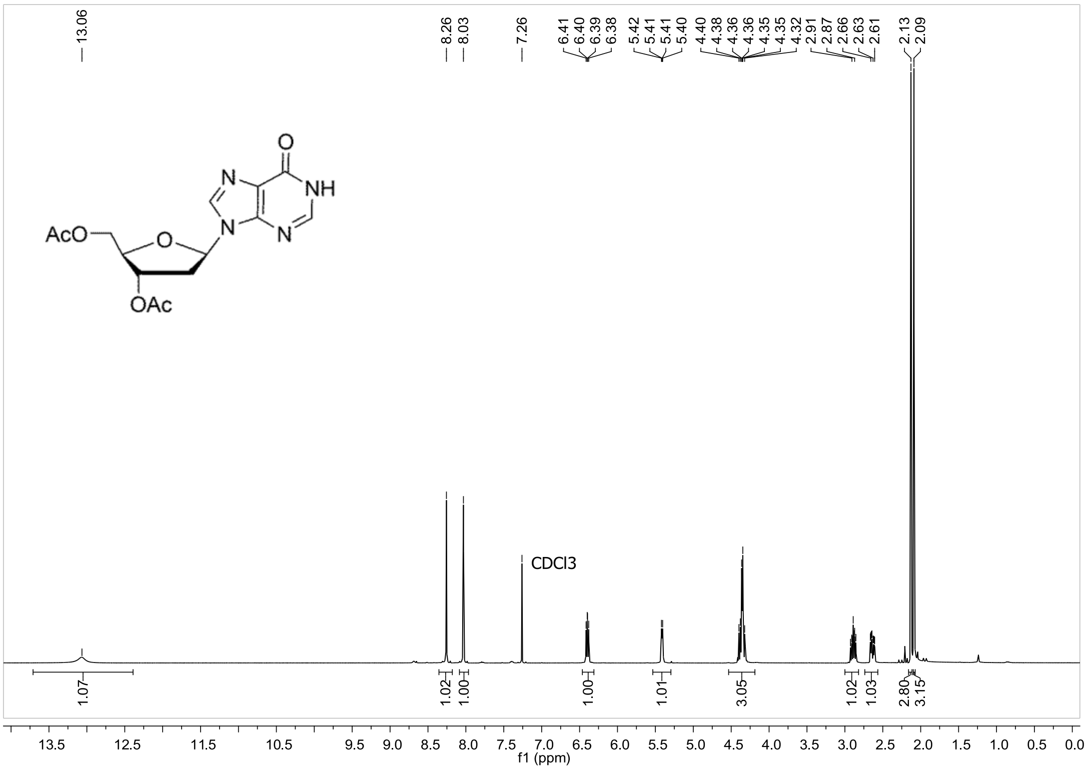

^{13}C NMR spectrum of 1

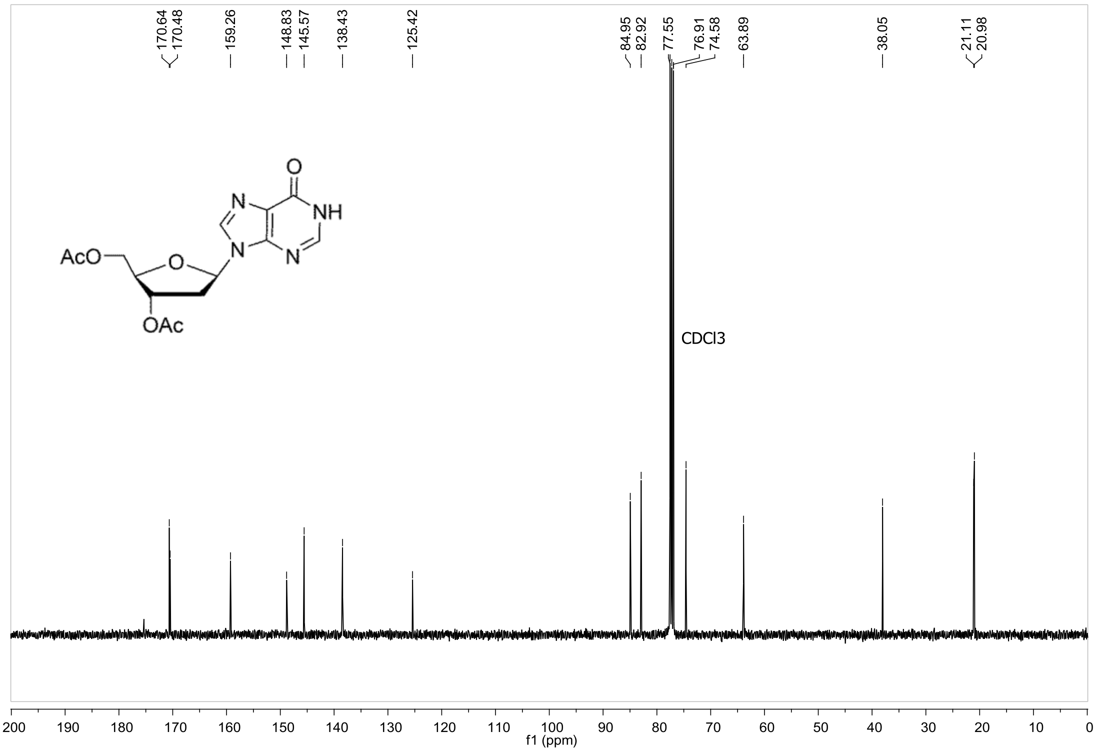

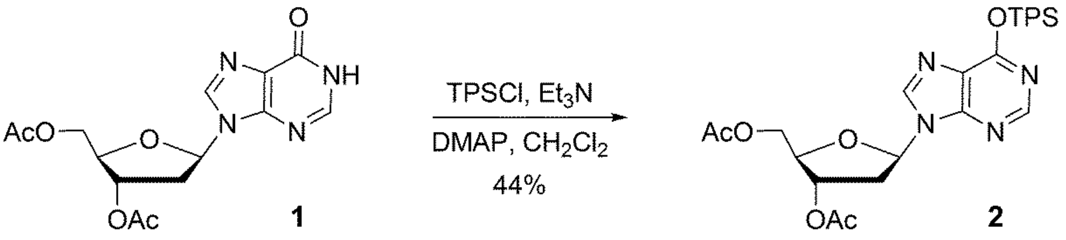

Compound 2. To a solution of **1** (400.0 mg, 1.19 mmol) in CH_2Cl_2 was added 4-DMAP (14.5 mg, 0.12 mmol), Et_3N (0.668 mL, 4.76 mmol) and triisopropylsulfonyl chloride (720.9 mg, 2.38 mmol) and the resulting mixture was stirred at 22 °C for 50 min. The solvent was removed *in vacuo* and the residue was purified by flash silica gel column chromatography using a gradient elution of (Pet. ether:EtOAc; 95:5 to 85:15) to give compound **2** as a white solid (315.0 mg, 44% yield).

^{1}H NMR (400 MHz, $CDCl_3$): δ 8.58 (s, 1H), 8.23 (s, 1H), 7.22 (s, 2H), 6.47 (dd, J = 7.7, 6.1 Hz, 1H), 5.44 (dd, J = 4.0, 2.3 Hz, 1H), 4.48 – 4.26 (m, 5H), 3.06 – 2.86 (m, 2H), 2.65 (ddd, J = 14.2, 6.0, 2.6 Hz, 1H), 2.14 (s, 3H), 2.07 (s, 3H), 1.39 – 1.12 (m, 18H).

^{13}C NMR (101 MHz, $CDCl_3$): δ 170.54, 170.45, 155.22, 154.68, 153.79, 151.81, 151.34, 142.96, 131.32, 124.19, 123.61, 85.22, 83.03, 74.51, 63.83, 37.79, 34.52, 30.01, 24.77, 23.71, 21.10, 20.95.

HR-ESI-MS $(M + Na)^+$: calcd. for $C_{29}H_{38}N_4O_8SNa$ 625.2303, found 625.2251.

^{1}H NMR spectrum of 2

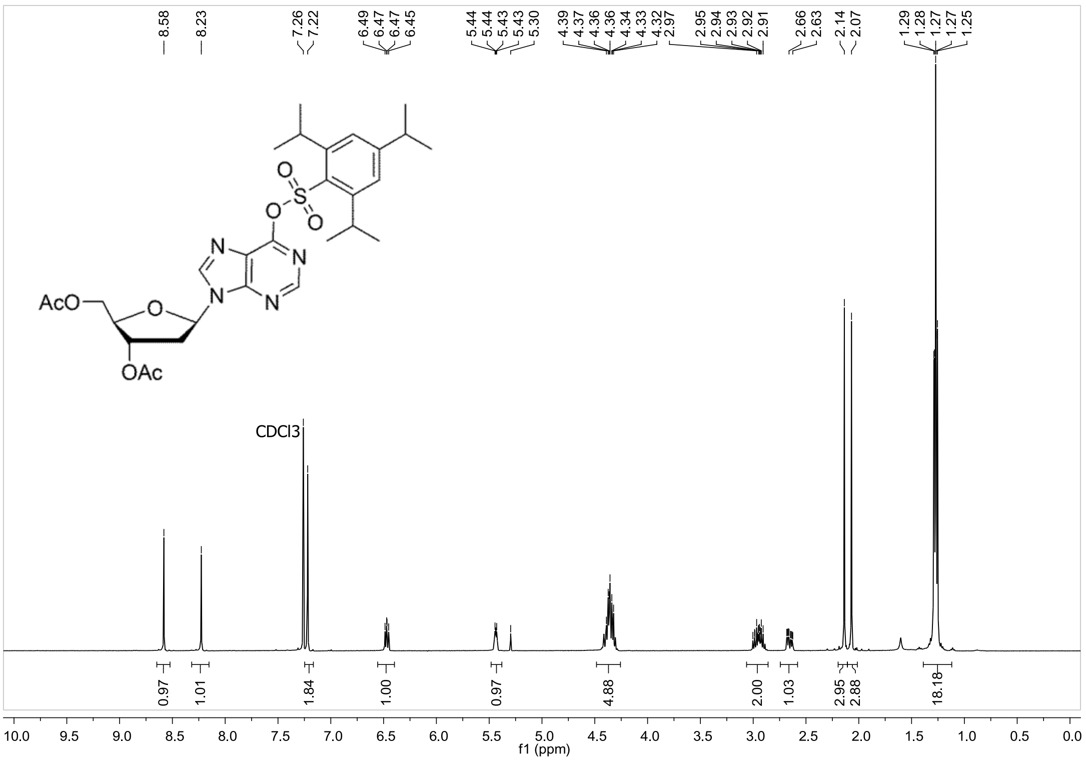

^{13}C NMR spectrum of 2

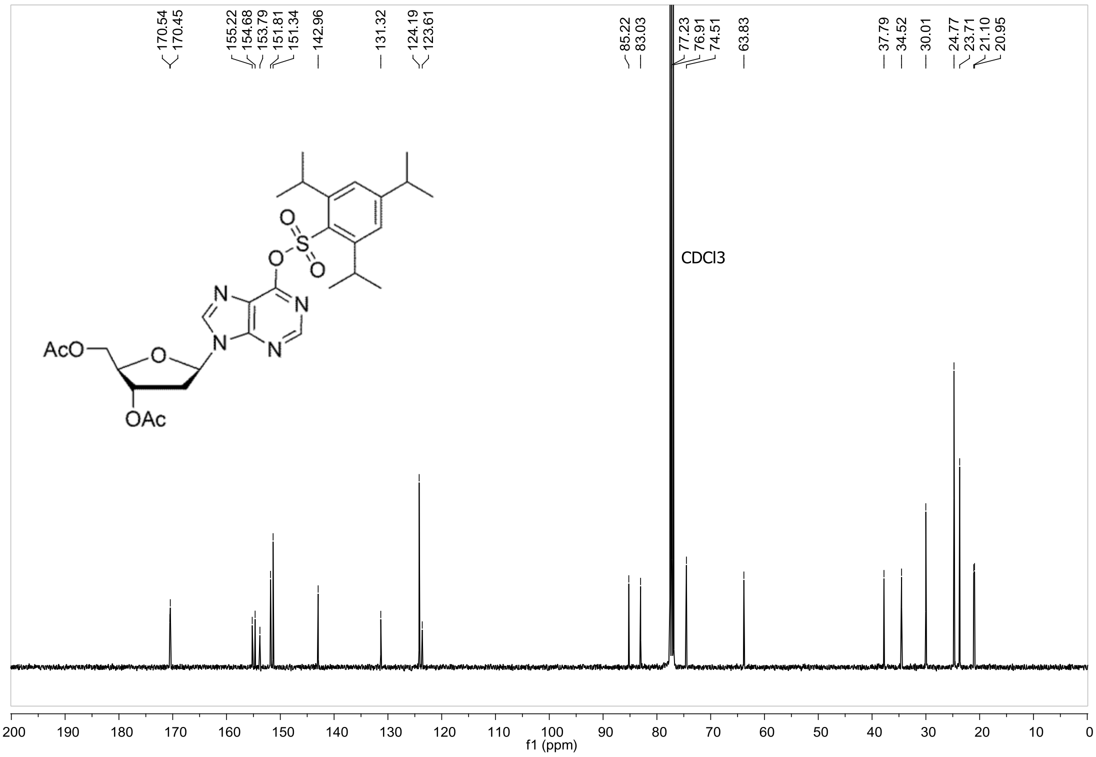

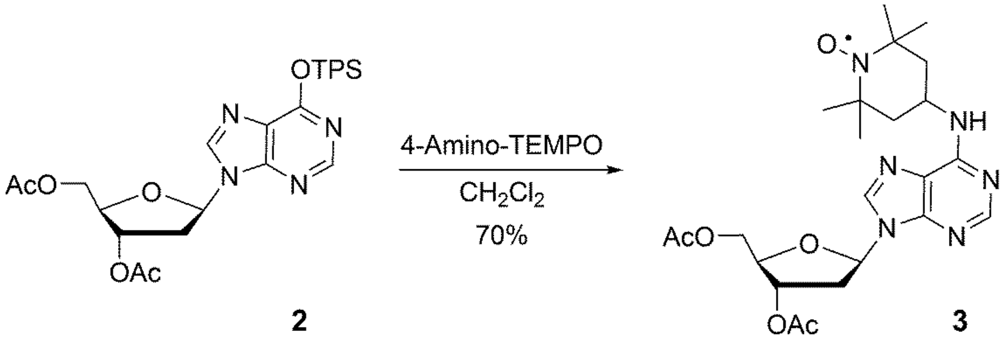

Compound 3. A solution of compound **2** (280.0 mg, 0.46 mmol) and 4-amino-TEMPO (164.7 mg, 0.96 mmol) in CH_2Cl_2 (2 mL) was refluxed for 3 days. The solvent was removed *in vacuo* and the crude solid was purified by flash silica gel column chromatography using a gradient elution of (CH_2Cl_2:MeOH; 98:2 to 95:5) to give compound **3** as light orange solid (160.0 mg, 70% yield).

^{1}H-NMR (400 MHz, $CDCl_3$): δ 8.57 (br s), 8.09 (br s), 8.09 (br s), 6.60 (br s), 5.59 (br s), 5.42 (br s), 4.50 (br s), 3.11 (br s), 2.78 (br s), 2.29 (br s), 2.25 (br s).

^{13}C NMR (101 MHz, $CDCl_3$): δ 167.59, 167.47, 152.53, 151.13, 135.85, 81.83, 79.73, 71.78, 61.19, 34.95, 18.40, 18.28.

HR-ESI-MS $(M + H)^+$: calcd. for $C_{23}H_{34}N_6O_6$ 490.2534, found 490.2503.

^{1}H NMR spectrum of 3

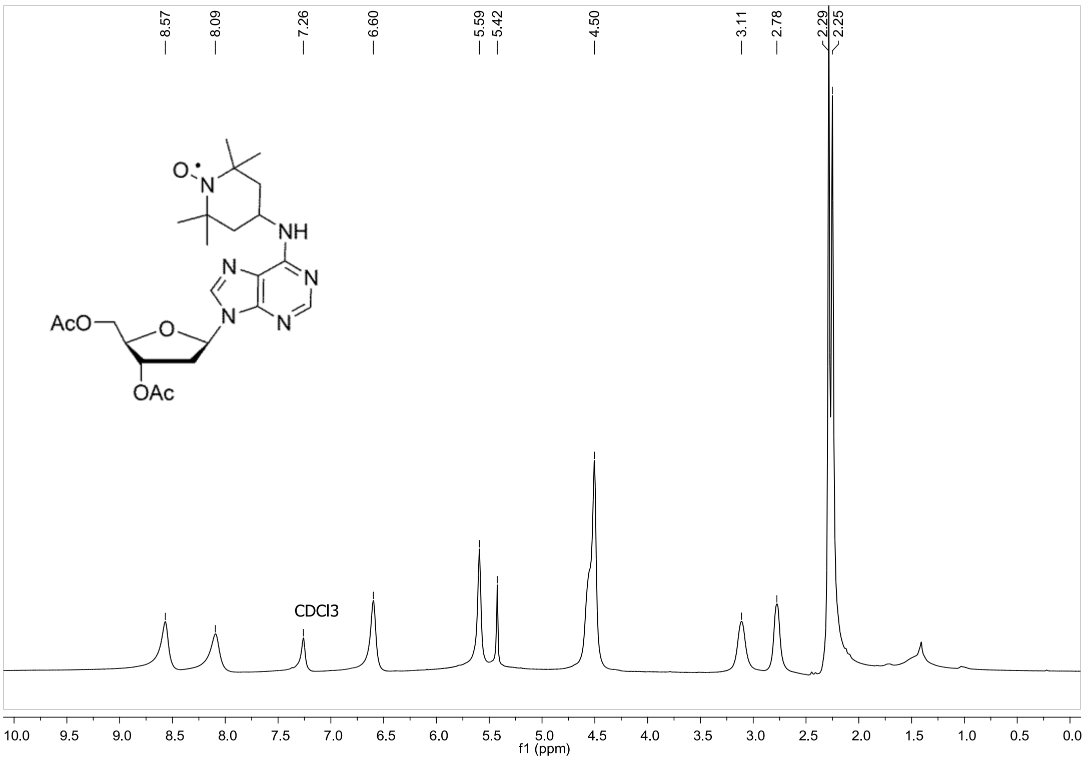

^{13}C NMR spectrum of 3

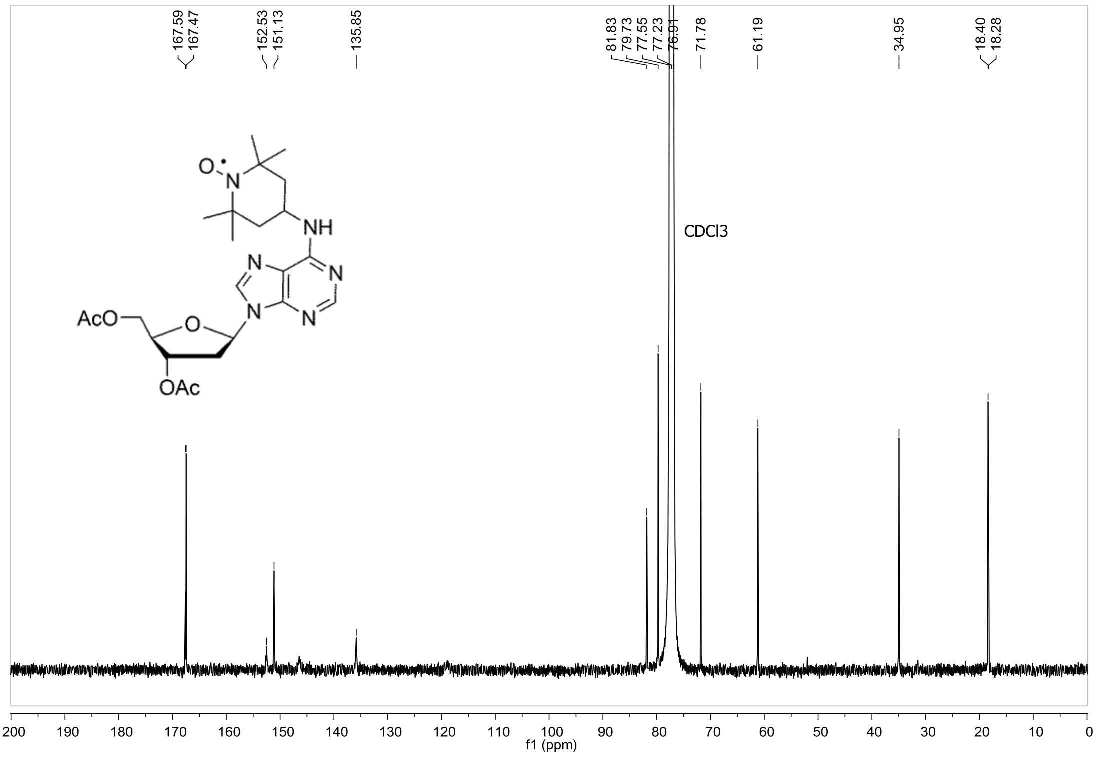

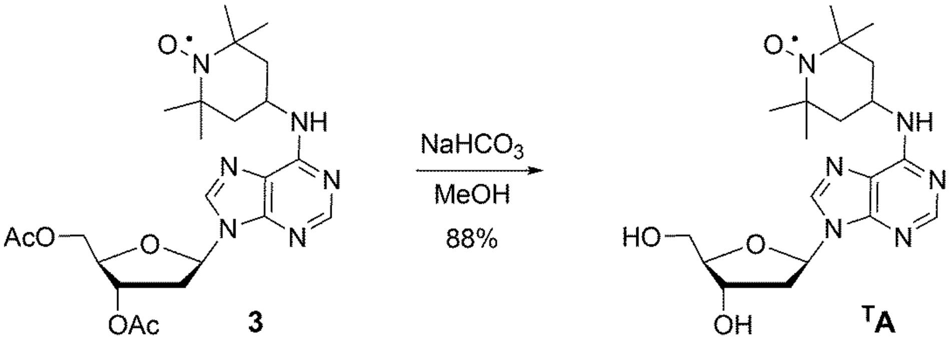

Spin-labeled nucleoside ^{T}A. To a solution of **3** (130.0 mg, 0.27 mmol) in MeOH (3.0 mL) was added $NaHCO_3$ (223.2 mg, 2.7 mmol) and the resulting mixture stirred at 22 °C for 19 h. The catalyst was filtered off, filtrate was evaporated *in vacuo* and the crude solid was purified by flash silica gel column chromatography using a gradient elution of (CH_2Cl_2:MeOH; 95:5 to 90:10) to give **^{T}A** as light orange solid (95.0 mg, 88% yield).

^{1}H NMR (400 MHz, $CDCl_3$): δ 8.53 (br s), 7.96 (br s), 6.76 (br s), 6.49 (br s), 5.43 (br s), 5.23 (br s), 4.21 (br s), 3.97 (br s), 3.38 (br s), 2.72 (br s), 2.33 (br s), 1.42 (br s), 1.22 (br s).

^{13}C NMR (101 MHz, $CDCl_3$): δ 152.72, 150.28, 145.06, 138.26, 120.14, 88.65, 85.96, 70.64, 61.23, 43.21, 39.49, 8.38.

HR-ESI-MS $(M + H)^+$: calcd. for $C_{19}H_{30}N_6O_4$ 406.2323, found 406.2346.

^{1}H NMR spectrum of ^{T}A

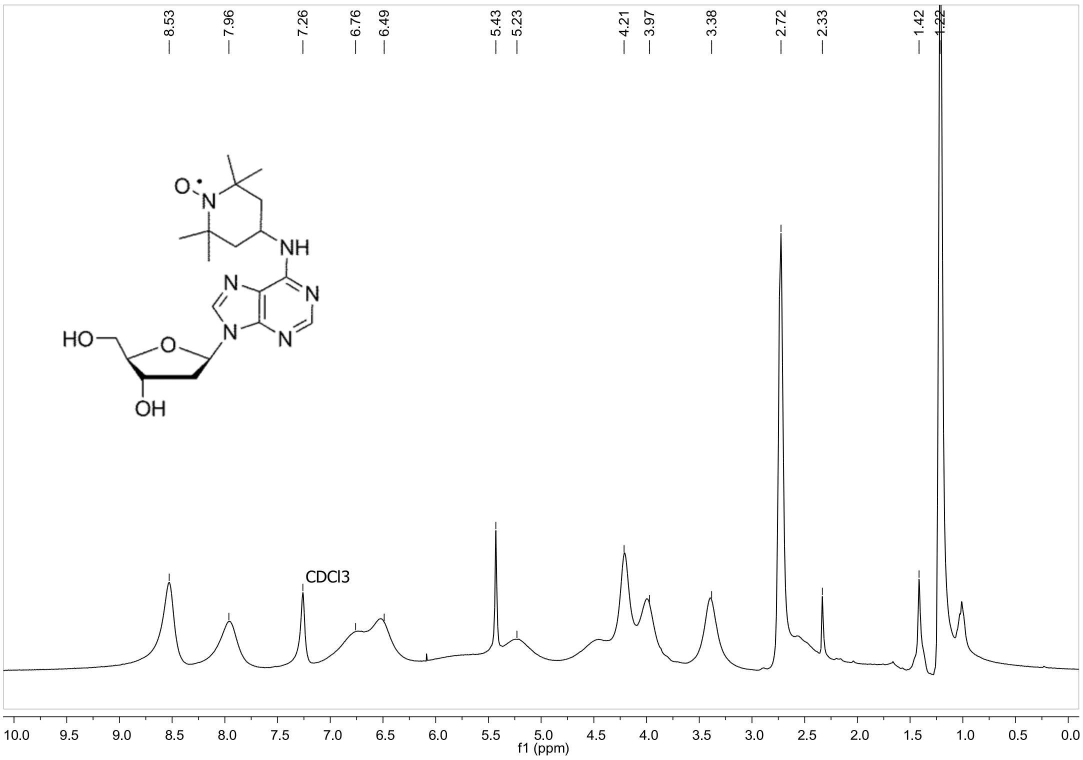

^{13}C NMR spectrum of ^{T}A

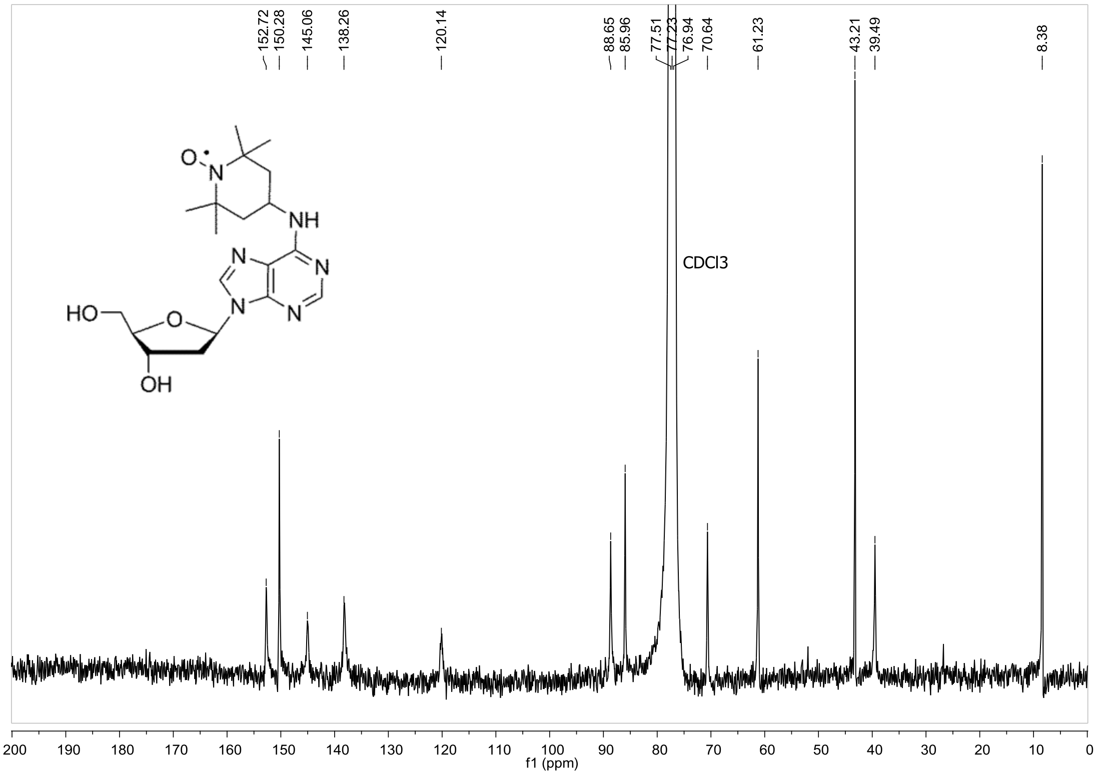

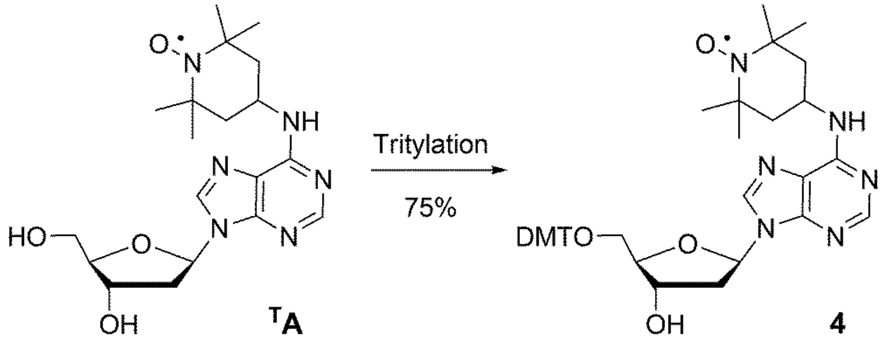

DMT-* *^{T}A (4). **^{T}A** (95.0 mg, 0.23 mmol), DMTr-Cl (142.9 mg, 0.42 mmol) and 4-DMAP (2.9 mg, 0.023 mmol) were place in a round bottom flask and kept under vacuum for 20 h. Pyridine (2.4 mL) was added and the resulting solution was stirred at 22 °C for 3 h. MeOH (100 µL) was added and the solvent was removed under reduced pressure to give crude. The crude solid was purified by flash silica gel column chromatography using a gradient elution of (CH_2Cl_2:MeOH; 100:0 to 97.5:2 + 0.5% Et_3N) on a column prepared in 99.5% CH_2Cl_2 + 0.5% Et_3N. **DMT-^{T}A** (**4**) was obtained as an orange solid (124.0 mg, 75% yield).

^{1}H NMR (400 MHz, $CDCl_3$): δ 8.32 (br s), 7.91 (br s), 7.37 (br s), 7.16 (br s), 6.77 (br s), 6.45 (br s), 5.25 (br s), 4.78 (br s), 4.17 (br s), 3.75 (br s), 3.39 (br s), 2.79 (br s), 2.56 (br s), 2.54 (br s), 2.53 (br s), 1.02 (br s).

^{13}C NMR (101 MHz, $CDCl_3$): δ 157.50, 152.75, 143.44, 134.61, 134.56, 129.02, 127.10, 126.96, 125.99, 112.23, 85.51, 83.31, 71.42, 62.80, 54.51, 52.94, 45.10, 39.85, 10.19.

HR-ESI-MS $(M + H)^+$: calcd. for $C_{40}H_{48}N_6O_6$ 708.3630, found 708.3601.

^{1}H NMR spectrum of DMT- ^{T}A

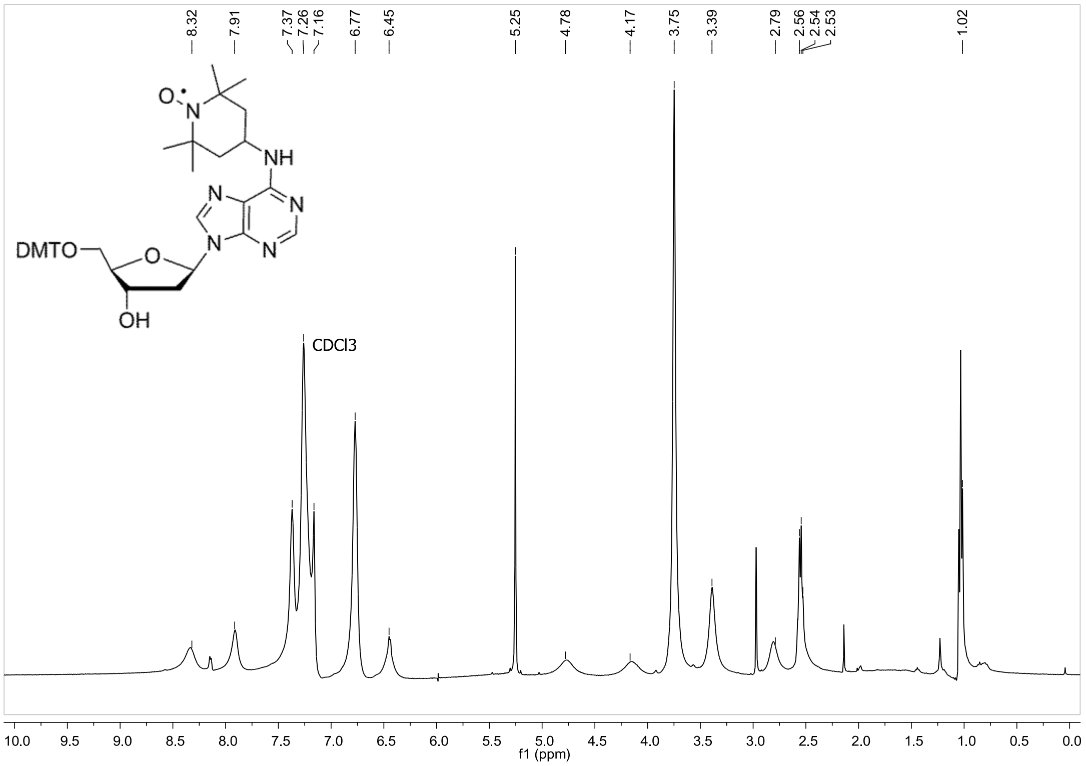

^{13}C NMR spectrum of DMT- ^{T}A

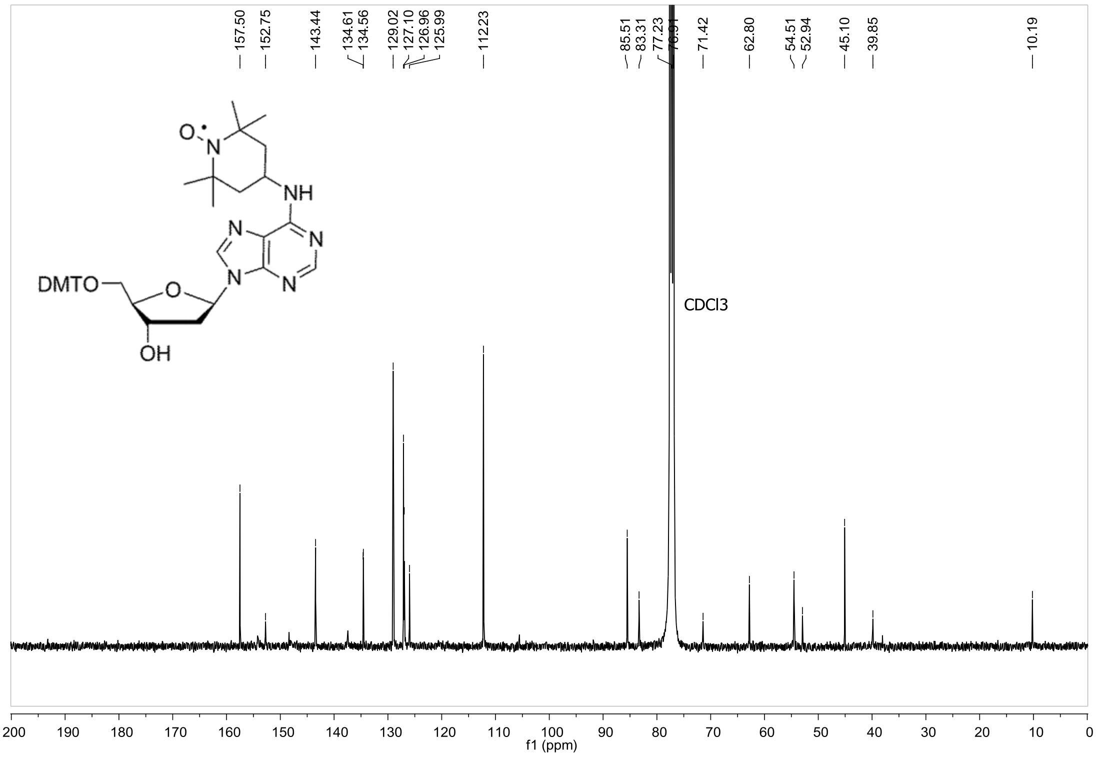

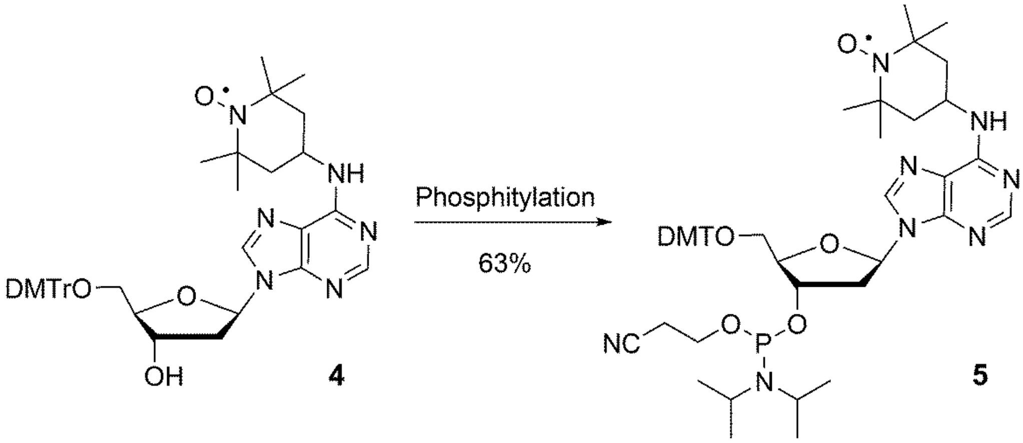

^{T}A phosphoramidite (5). **DMT-^{T}A** (**4**) (50.0 mg, 0.071 mmol) and diisopropylammonium tetrazolide (15.6 mg, 0.092 mmol) were dissolved in pyridine (2 mL) and the pyridine removed *in vacuo*. The residue was kept *in vacuo* for 19 h, followed by dissolution in CH_2Cl_2 (2.5 mL) and addition of 2-cyanoethyl *N*,*N*,*N'*,*N'*-tetraisopropyl phosphoramidite (27.7 mg, 0.092 mmol). The reaction mixture was stirred at 22 °C for 3 h, diluted with CH_2Cl_2 (10 mL) and washed successively with saturated aq. $NaHCO_3$ (3 x 10 mL) and saturated aq. NaCl (3 x 10 mL). The organic solution was dried over anhydrous Na_2SO_4, filtered and concentrated *in vacuo* to give a crude solid. The solid was purified by precipitation by first dissolving it in diethyl ether (2 mL) followed by precipitation with n-hexane (80 mL). The liquid was decanted and the operation repeated twice to furnish phosphoramidite **5** as an orange solid (41.0 mg, 63% yield).

^{1}H NMR (400 MHz, $CDCl_3$): δ 8.34 (br s), 7.95 (br s), 7.37 (br s), 6.76 (br s), 6.43 (br s), 5.26 (br s), 4.74 (br s), 4.26 (br s), 3.57 (br s), 3.44 (br s), 2.80 (br s), 2.59 (br s), 2.43 (br s), 1.23 (br s), 1.18 (br s), 1.16 (br s), 1.08 (br s), 0.05 (br s).

^{31}P NMR (162 MHz, $CDCl_3$): δ 148.88, 148.80, 14.35.

HR-ESI-MS $(M + H)^+$: calcd. for $C_{49}H_{65}N_8O_7P$ 908.4708, found 908.4766.

^{1}H NMR spectrum of 5

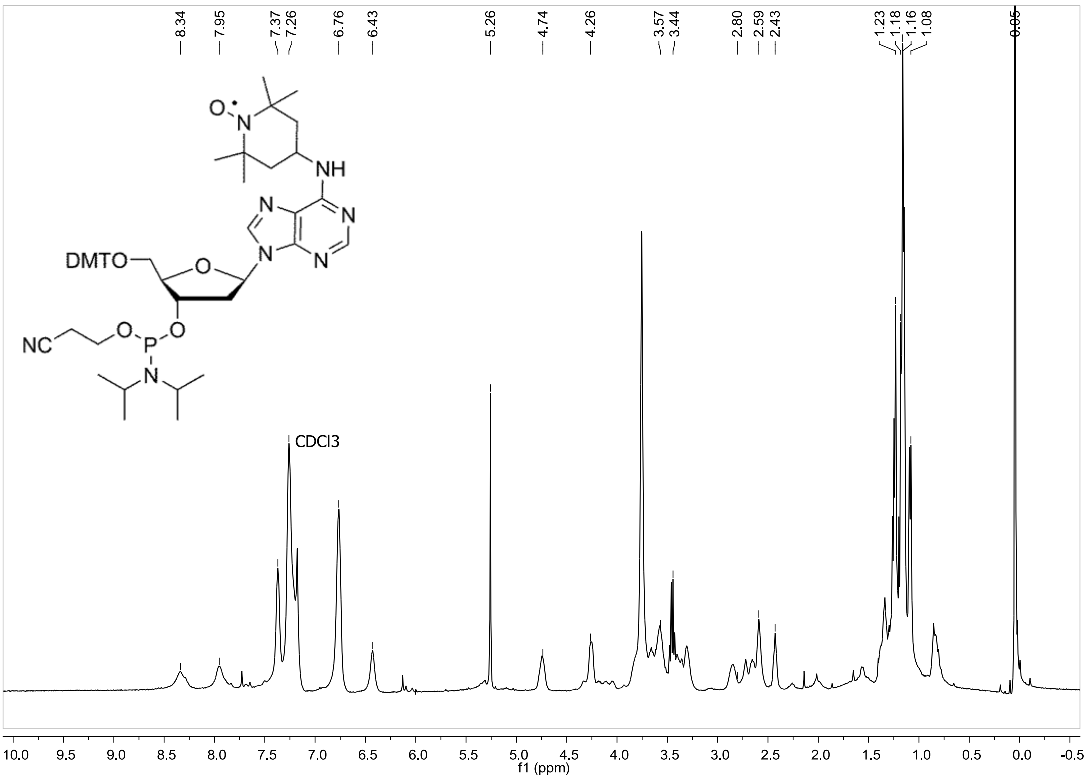

^{31}P NMR spectrum of 5

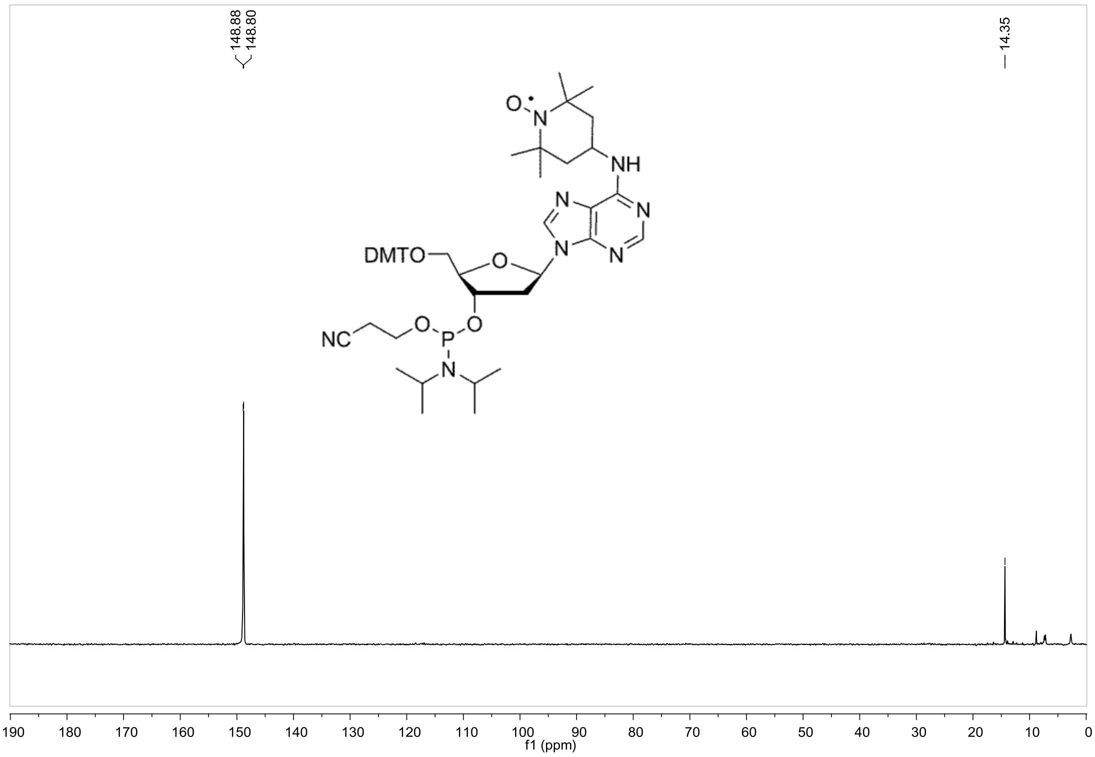

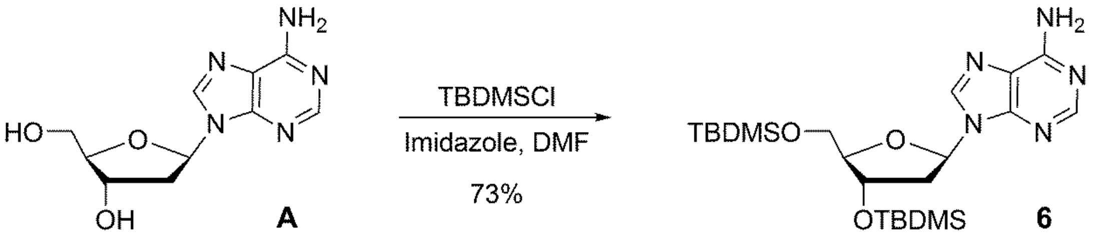

Compound 6. To a solution of 2′-deoxyadenosine (1.00 g, 3.98 mmol) in DMF (5.00 mL) was added *tert*-butyldimethylsilyl chloride (2.40 g, 15.9 mmol) and imidazole (2.16 g, 31.8 mmol). The resulting reaction mixture was stirred at 22 °C for 3 h, after which the solvent was removed *in vacuo*. The crude product was purified by flash silica gel column chromatography using a gradient elution of (CH_2Cl_2:MeOH; 100:0 to 95:5) to give **6** as a white solid (1.4 gm, 73% yield).

^{1}H NMR (400 MHz, $CDCl_3$): δ 8.34 (s, 1H), 8.13 (s, 1H), 6.45 (t, J = 6.4 Hz, 1H), 5.89 (s, 2H), 4.61 (dt, J = 5.8, 3.6 Hz, 1H), 4.00 (dd, J = 7.2, 3.3 Hz, 1H), 3.87 (dd, J = 11.2, 4.2 Hz, 1H), 3.76 (dd, J = 11.2, 3.2 Hz, 1H), 2.72 – 2.56 (m, 1H), 2.42 (ddd, J = 13.1, 6.1, 3.9 Hz, 1H), 0.90 (d, J = 1.1 Hz, 18H), 0.09 (d, J = 5.0 Hz, 12H).

^{13}C NMR (101 MHz, CDCl3): δ 155.70, 153.16, 149.82, 139.23, 120.26, 88.07, 84.51, 72.09, 62.98, 41.50, 26.16, 25.96, 18.63, 18.21, -4.46, -4.60, -5.17, -5.28.

HR-ESI-MS $(M + H)^+$: calcd. for $C_{22}H_{42}N_5O_3Si_2$ 480.2821, found 480.2848.

^{1}H NMR spectrum of 6

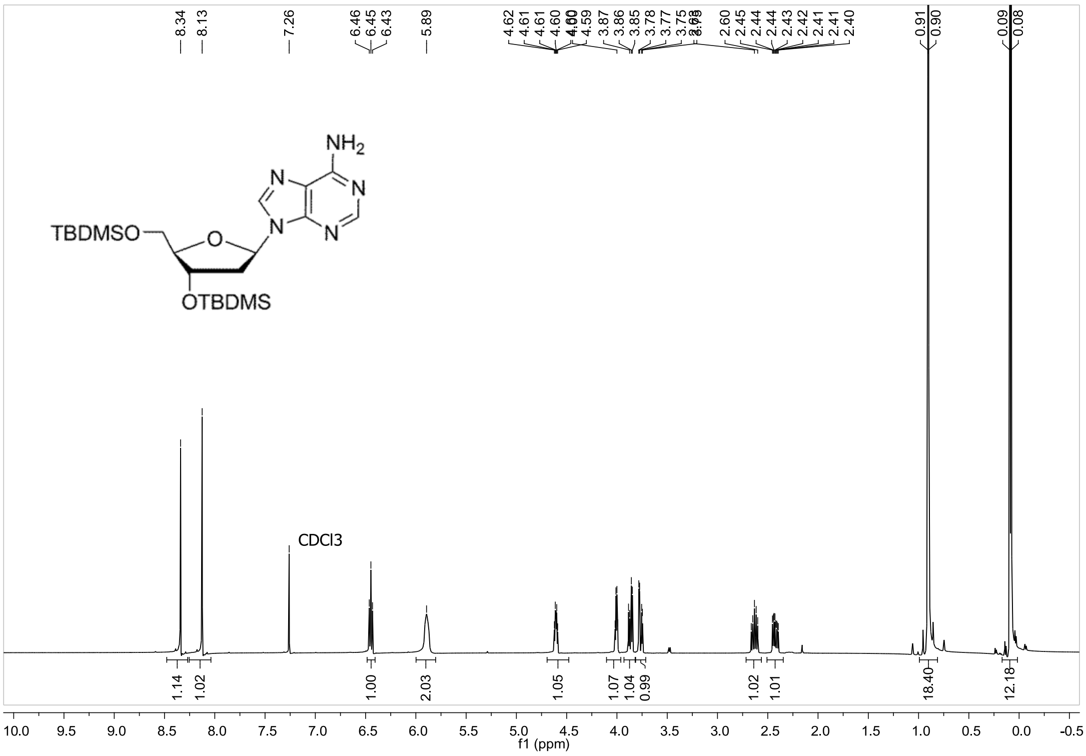

^{13}C NMR spectrum of 6

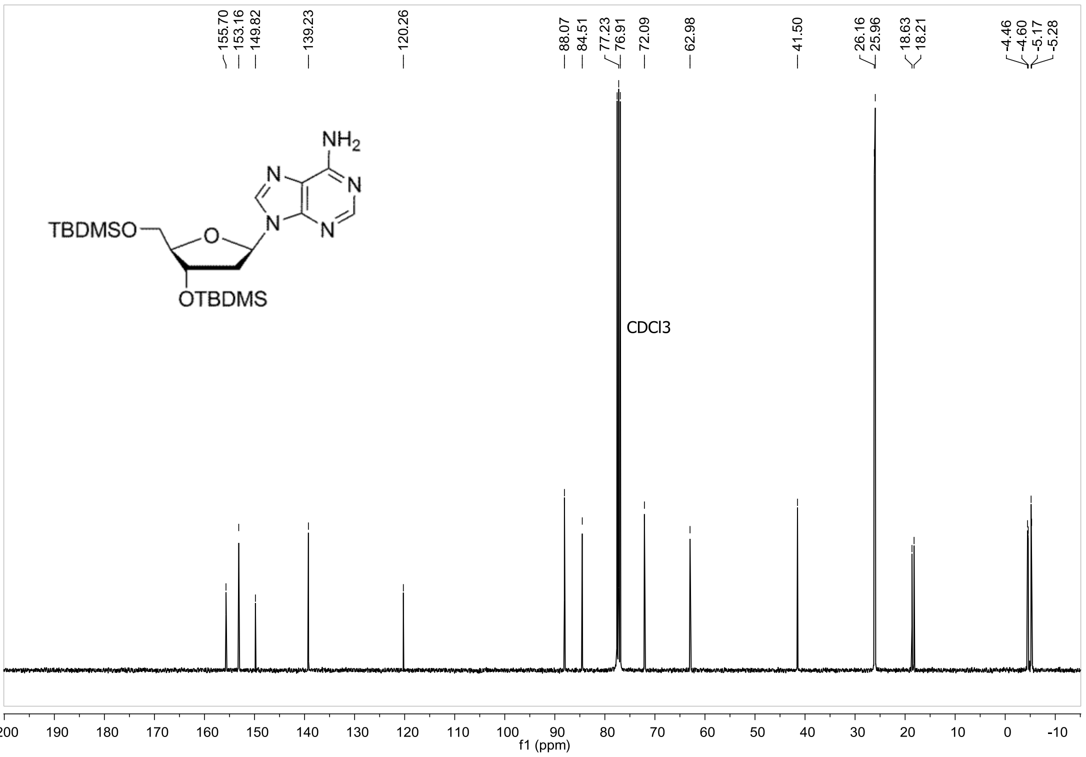

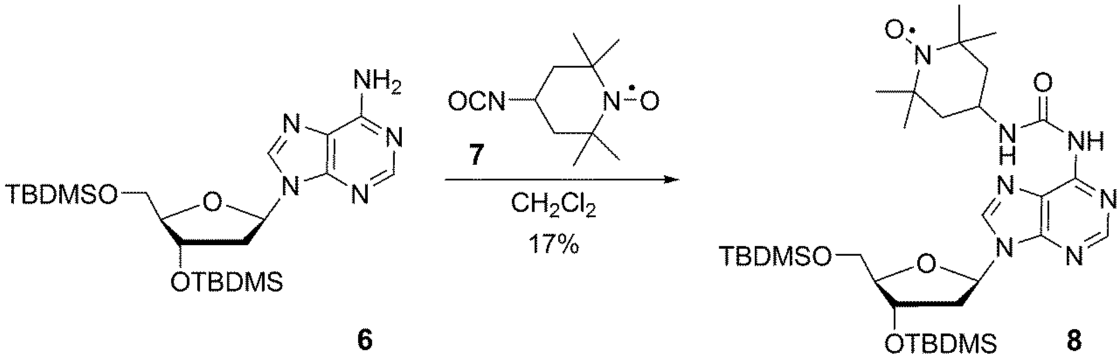

Compound 8. To a solution of compound **6** (50.0 mg, 0.10 mmol) in dichloromethane (2.0 mL) was added 4-isocyanato-TEMPO (20.0 mg, 0.10 mmol) and the resulting mixture was stirred for 3 days at 40 °C. Another 1.0 equivalent of 4-isocyanato-TEMPO (20.0 mg, 0.10 mmol) was added and stirred reaction for another 16 h at 40 °C. The solvent was removed *in vacuo* and the crude product was purified by flash silica gel column chromatography using a gradient elution (CH_2Cl_2:MeOH; 100:0 to 97:3) to give compound **8** as an orange solid (12.0 mg, 17% yield).

^{1}H NMR (400 MHz, $CDCl_3$): δ 9.32 (br s), 9.16 (br s), 7.26 (br s), 6.02 (br s), 5.40 (br s), 4.80 (br s), 4.64 (br s), 4.56 (br s), 3.42 (br s), 3.24 (br s), 2.80 (br s), 2.02 (br s), 1.68 (br s), 0.85 (br s).

^{13}C NMR (101 MHz, $CDCl_3$): δ 150.87, 147.28, 146.15, 146.07, 137.84, 116.94, 84.21, 80.79, 67.94, 58.91, 37.58, 25.83, 22.16, 21.96, 14.51, 14.10, 10.37, -8.41, -8.56, -9.17, -9.25.

HR-ESI-MS $(M + Na)^+$: calcd. for $C_{32}H_{58}N_7O_5Si_2N_a$ 699.4038, found 699.4355.

^{1}H NMR spectrum of 8

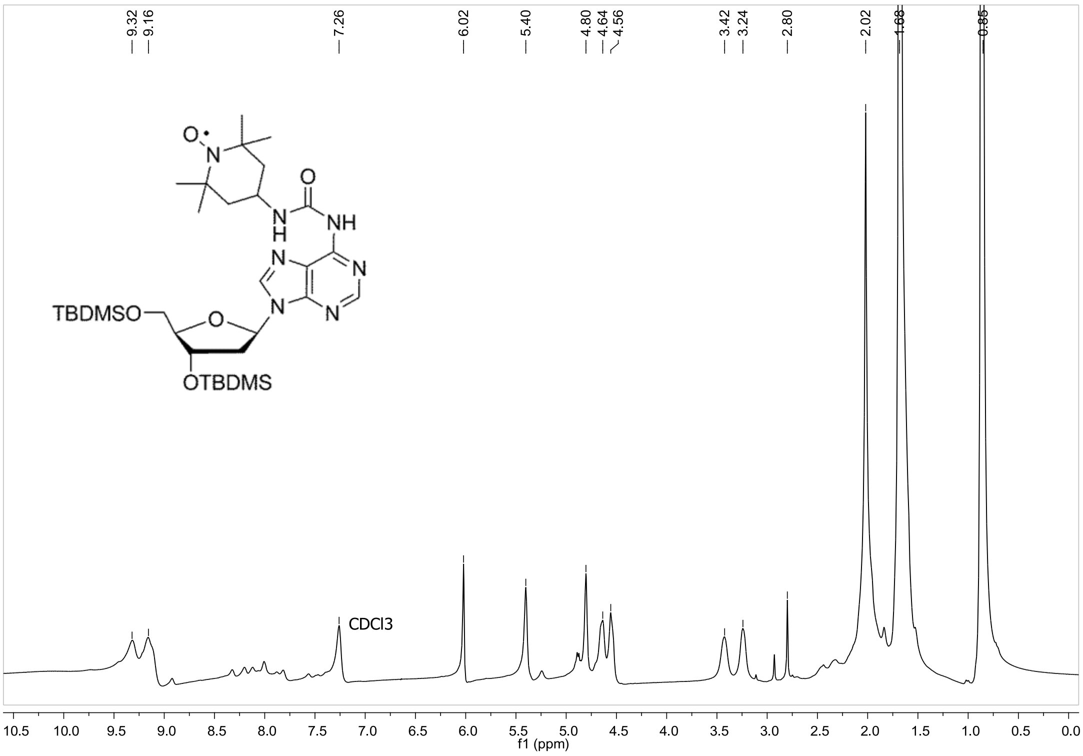

^{13}C NMR spectrum of 8

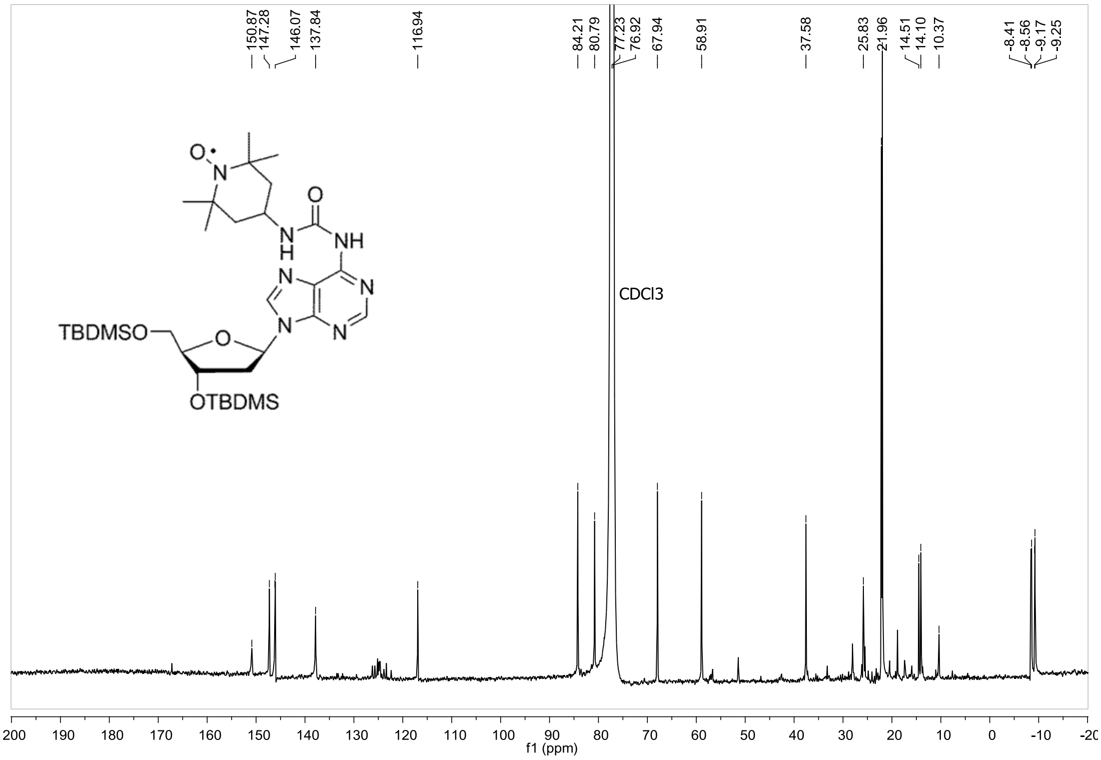

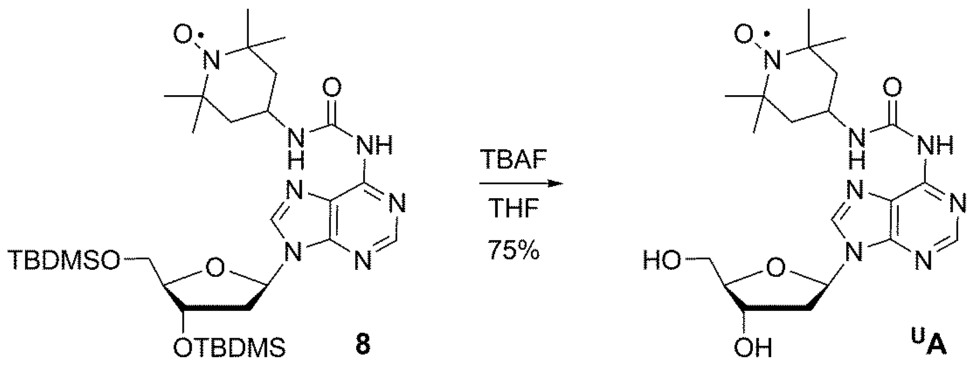

Spin-labeled nucleoside ^{U}***A.*** To a solution of compound **8** (150.0 mg, 0.22 mmol) in THF (2 mL) was added tetrabutylammonium fluoride (0.128 mL, 0.46 mmol) and the resulting mixture was stirred at 22 °C for 16 h. The solvent was removed *in vacuo* to give a brownish solid. The crude product was purified by flash silica gel column chromatography using a gradient elution (CH_2Cl_2:MeOH; 100:0 to 97:3) to give compound ^{U}**A** as an orange solid (75.0 mg, 75% yield).

^{1}H NMR (400 MHz, $CDCl_3$): δ 9.00 (br s), 8.09 (br s), 6.66 (br s), 6.14 (br s), 4.84 (br s), 3.70 (br s), 2.59 (br s), 2.16 (br s), 1.61 (br s), 0.83 (br s), 0.65 (br s).

^{13}C NMR (101 MHz, $CDCl_3$): δ 151.55, 147.28, 147.05, 146.00, 140.90, 118.27, 87.34, 84.28, 68.99, 60.39, 51.61, 42.65, 38.88, 27.64, 7.69.

HR-ESI-MS $(M + Na)^+$: calcd. for $C_{20}H_{30}N_7O_5Na$ 471.2201, found 471.2195.

^{1}H NMR spectrum of [U]A

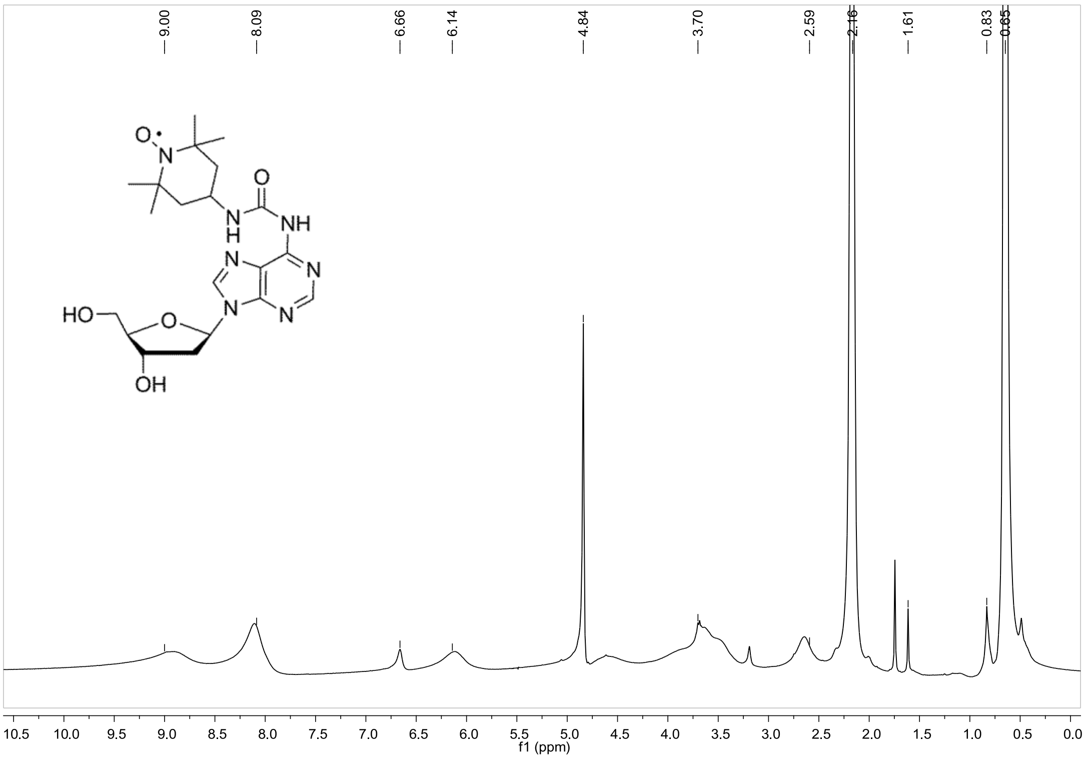

^{13}C NMR spectrum of [U]A

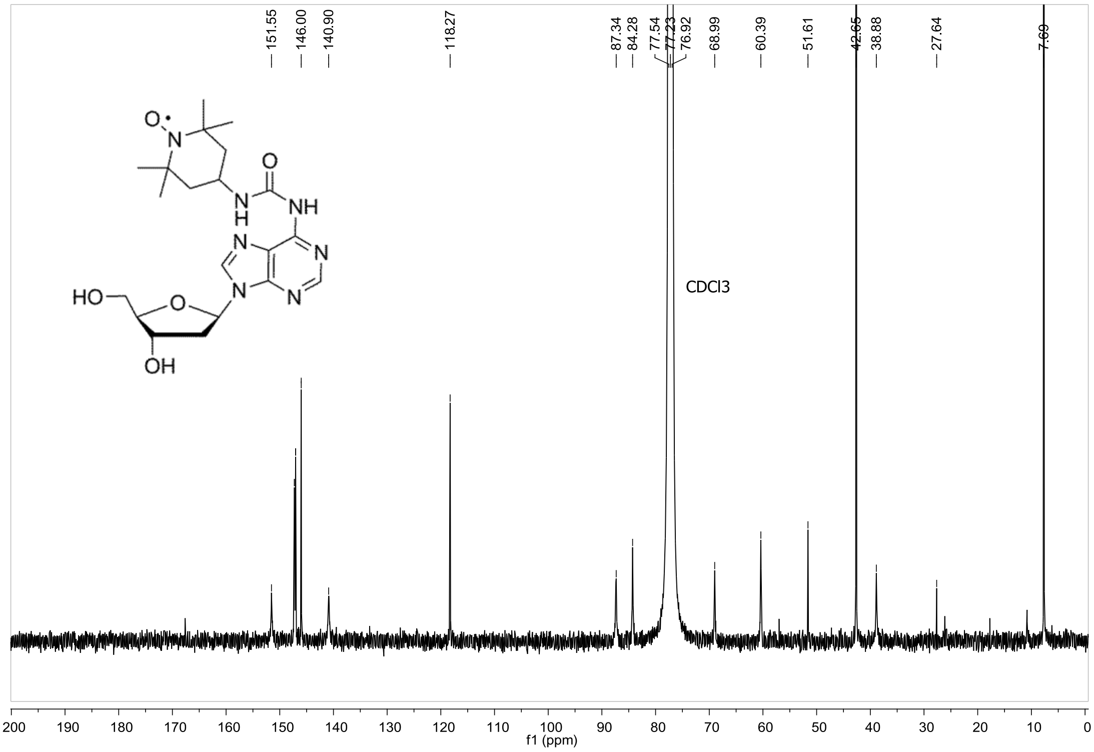

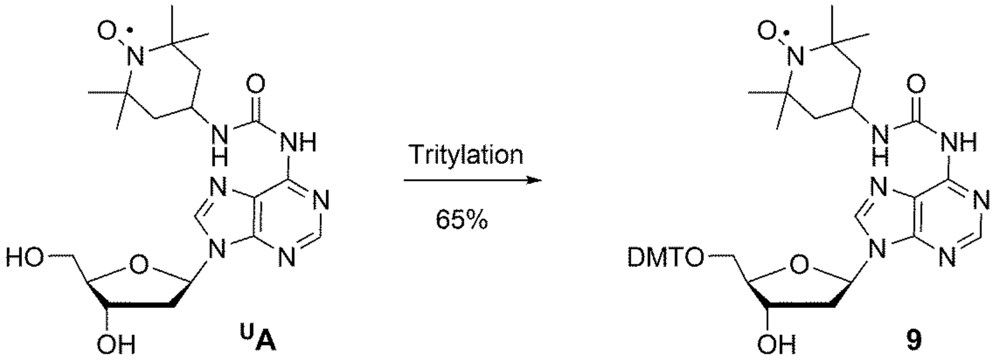

^{U}A 9

DMT- ^{U}A (9). **^{U}A** (70.0 mg, 0.16 mmol), DMTCl (95.2 mg, 0.28 mmol) and 4-DMAP (2.9 mg, 0.016 mmol) were placed in a round bottom flask and kept under vacuum for 20 h. Pyridine (1.8 mL) was added and the resulting solution was stirred at 22 °C for 3 h, after which MeOH (100 μL) was added. The solvent was removed *in vacuo* and the crude solid was purified by flash silica gel column chromatography using a gradient elution of (CH_2Cl_2:MeOH; 100:0 to 97.5:2 + 0.5% Et_3N), using a column prepared in 99.5% CH_2Cl_2 + 0.5% Et_3N, giving compound **DMT-^{U}A** (**9**) as an orange colored solid (76.0 mg, 65% yield).

^{1}H NMR (400 MHz, $CDCl_3$): δ 8.45 (br s), 8.15 (br s), 7.37 (br s), 6.76 (br s), 5.22 (br s), 3.73 (br s), 3.41 (br s), 2.97 (br s), 2.60 (br s), 1.26 (br s), 1.05 (br s), 0.84 (br s).

^{13}C NMR (101 MHz, $CDCl_3$): δ 154.81, 147.46, 146.25, 131.91, 131.88, 126.40, 124.46, 124.30, 123.36, 117.18, 109.57, 82.77, 60.34, 51.98, 51.50, 42.44, 37.63, 30.42, 29.98, 25.36, 25.24, 23.92, 18.98, 18.65, 16.74, 15.79, 10.71, 10.45, 7.81, 7.30.

HR-ESI-MS $(M + Na)^+$: calcd. for $C_{41}H_{48}N_7O_7Na$ 773.3507, found 773.3517.

^{1}H NMR spectrum of 9

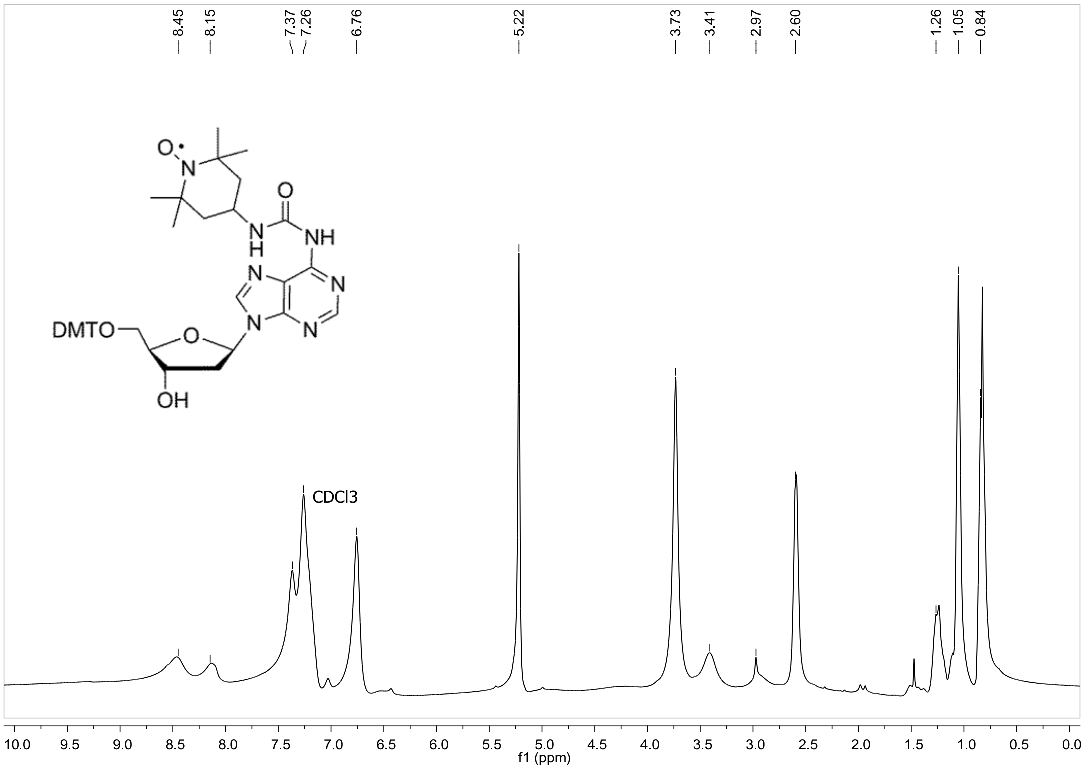

^{13}C NMR spectrum of 9

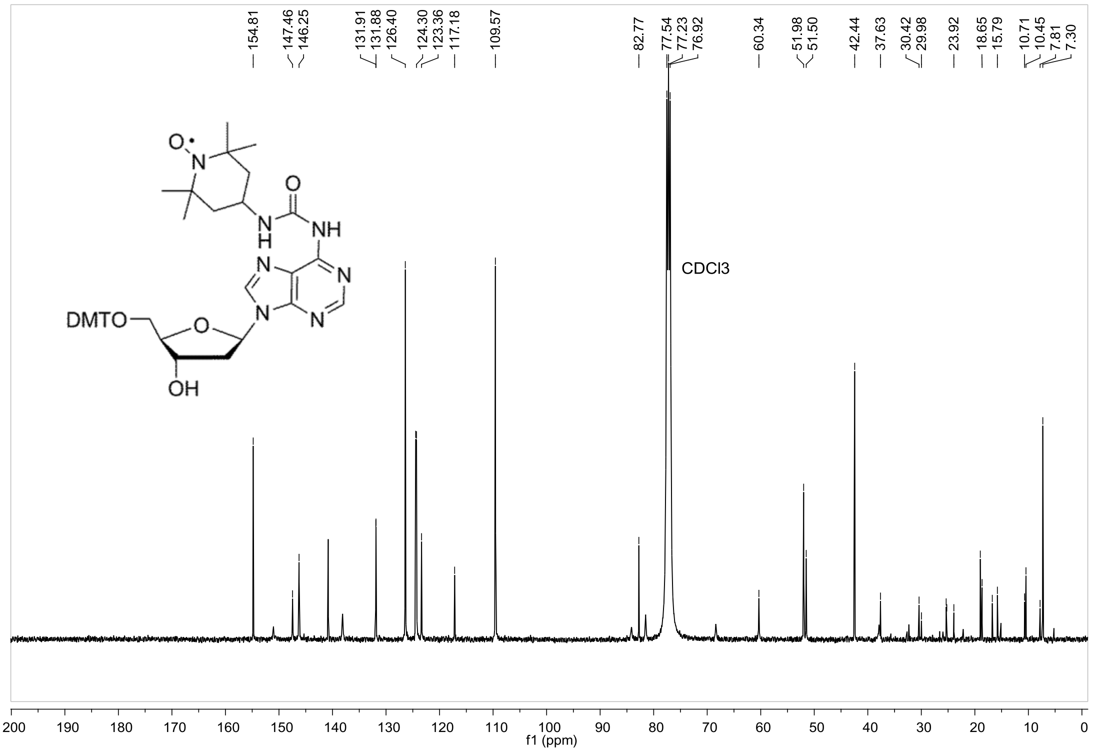

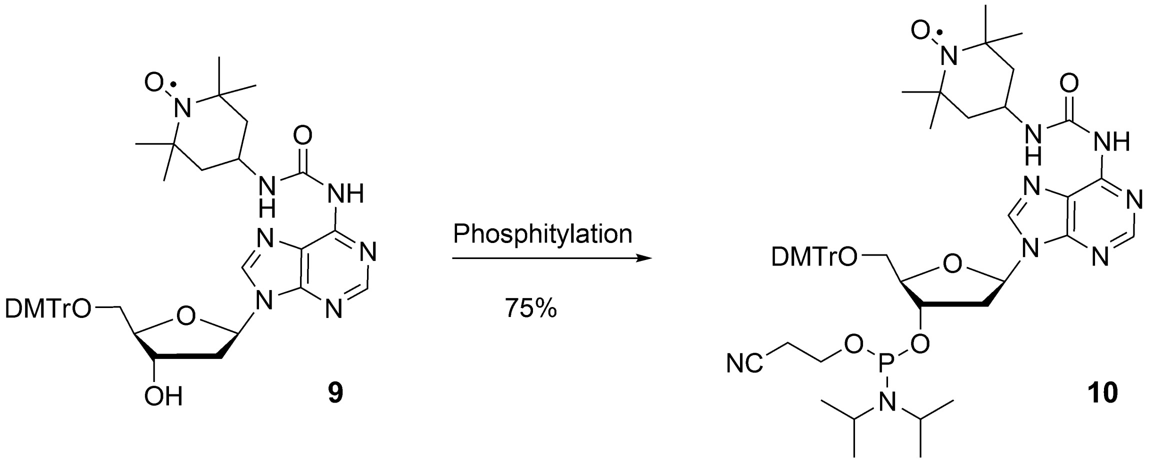

^{U}A phosphoramidite (10). **DMT-^{U}A** (**9**) (60.0 mg, 0.08 mmol) and diisopropylammonium tetrazolide (17.7 mg, 0.10 mmol) were dissolved in pyridine (2 mL) and the pyridine removed *in vacuo*. The residue was kept *in vacuo* for 19 h, followed by dissolution in CH_2Cl_2 (3.0 mL) and addition of 2-cyanoethyl *N*,*N*,*N*′,*N*′-tetraisopropyl phosphoramidite (31.3 mg, 0.10 mmol). The reaction mixture was stirred at 22 °C for 3 h, diluted with CH_2Cl_2 (10 mL) and washed successively with saturated aq. $NaHCO_3$ (3 x 10 mL) and saturated aq. NaCl (3 x 10 mL). The organic solution was dried over anhydrous Na_2SO_4, filtered and concentrated *in vacuo* to give a crude solid, which was purified by precipitation by first dissolving it in diethyl ether (1 mL), followed by addition of n-hexane (80 mL). The liquid was decanted and the operation repeated twice to furnish phosphoramidite **10** as an orange solid (75.0 mg, 75% yield).

^{1}H NMR (400 MHz, $CDCl_3$): δ 8.50 (br s), 8.12 (br s), 7.26 (br s), 6.77 (br s), 6.44 (br s), 5.26 (br s), 4.78 (br s), 4.28 (br s), 3.76 (br s), 3.60 (br s), 3.41 (br s), 3.34 (br s), 2.89 (br s), 2.68 (br s), 2.60 (br s), 2.45 (br s), 1.38 (br s), 1.36 (br s), 1.26 (br s), 1.24 (br s), 1.18 (br s), 1.17 (br s), 1.12 (br s), 1.10 (br s).

^{31}P NMR (162 MHz, $CDCl_3$): δ 149.07

HR-ESI-MS $(M + Na)^+$: calcd. for $C_{50}H_{65}N_9O_8PNa$ 973.4586, found 973.4546.

^{1}H NMR spectrum of 10

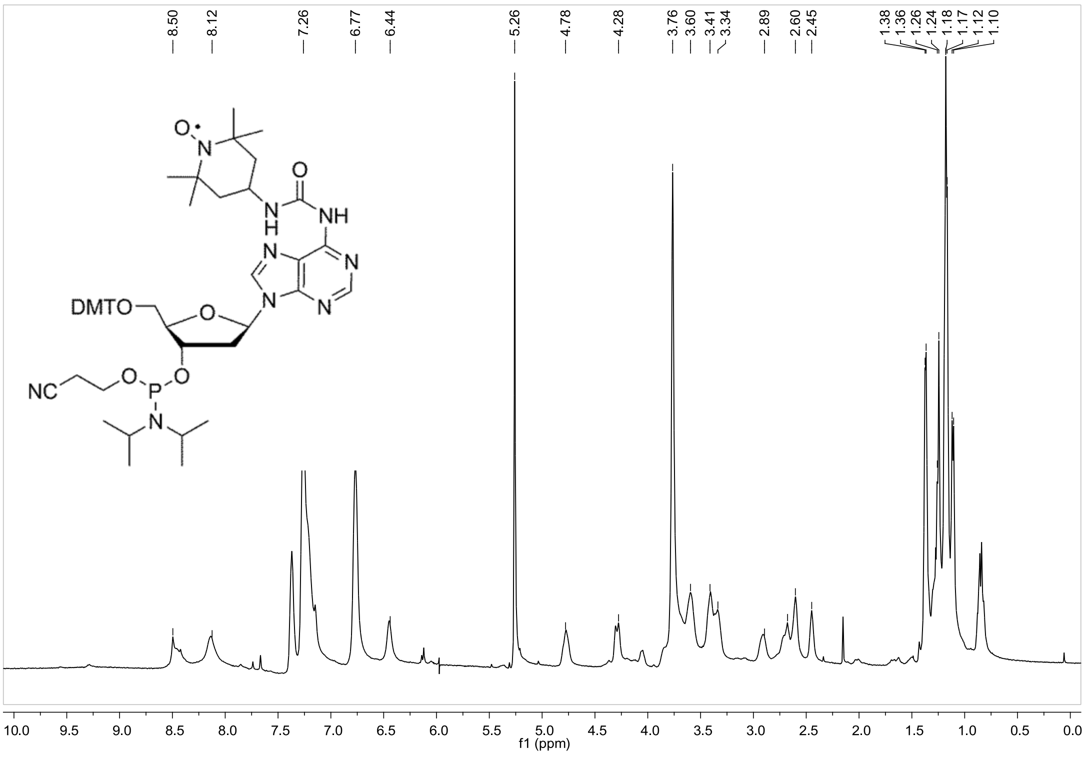

^{31}P NMR spectrum of 10

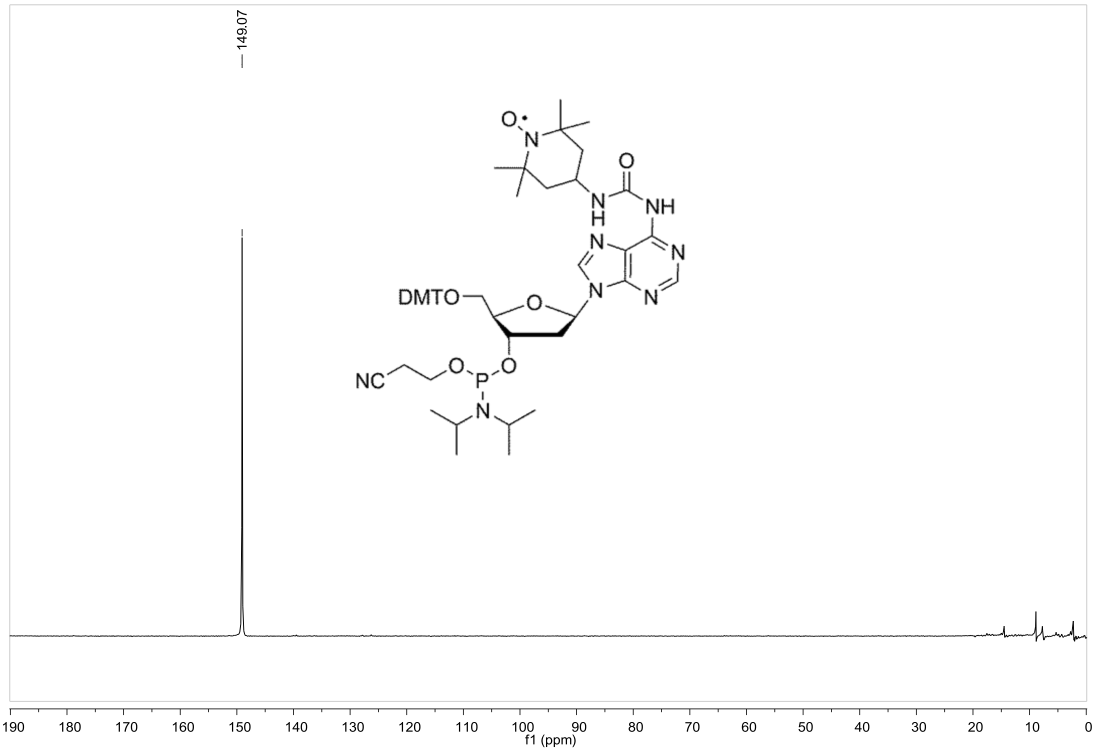

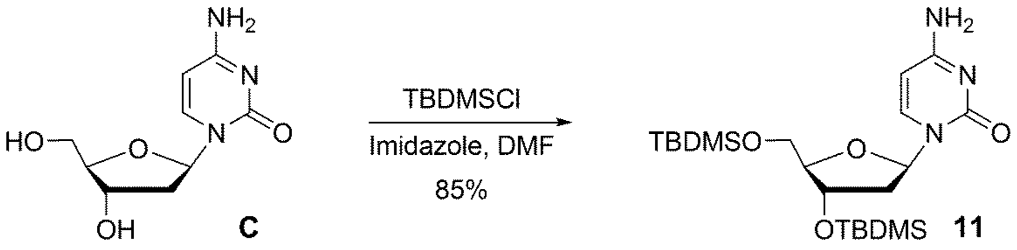

Compound 11. To a solution of 2′-deoxycytidine (1.0 g, 4.40 mol) in DMF (5 mL) was added *tert*-butyldimethylsilyl chloride (2.65 g, 17.6 mol) and imidazole (2.39 g, 35.1 mol). The reaction mixture was stirred at 22 °C for 3 h. The solvent was removed *in vacuo* and the crude product was purified by flash silica gel column chromatography, using a gradient elution (CH_2Cl_2:MeOH; 98:2 to 94:6) to give compound **11** as a white solid (1.7 g, 85% yield).

^{1}H NMR (400 MHz, $CDCl_3$): δ 7.97 (d, *J* = 7.4 Hz, 1H), 6.33 – 6.19 (m, 1H), 5.66 (d, *J* = 7.4 Hz, 1H), 4.36 (dd, *J* = 10.6, 5.8 Hz, 1H), 3.97 – 3.85 (m, 2H), 3.81 – 3.69 (m, 1H), 2.54 – 2.33 (m, 1H), 2.13 – 2.00 (m, 1H), 0.89 (d, *J* = 18.3 Hz, 18H), 0.07 (dd, *J* = 19.4, 1.2 Hz, 12H).

^{13}C NMR (101 MHz, $CDCl_3$): δ 165.78, 155.97, 141.60, 93.89, 87.49, 86.14, 70.48, 62.16, 42.41, 26.11, 25.93, 19.64, 18.56, 18.16, -4.36, -4.72, -5.26, -5.33.

HR-ESI-MS $(M + H)^+$: calcd. for $C_{21}H_{42}N_3O_4Si_2$ 456.2708, found 456.2716.

^{1}H NMR spectrum of 11

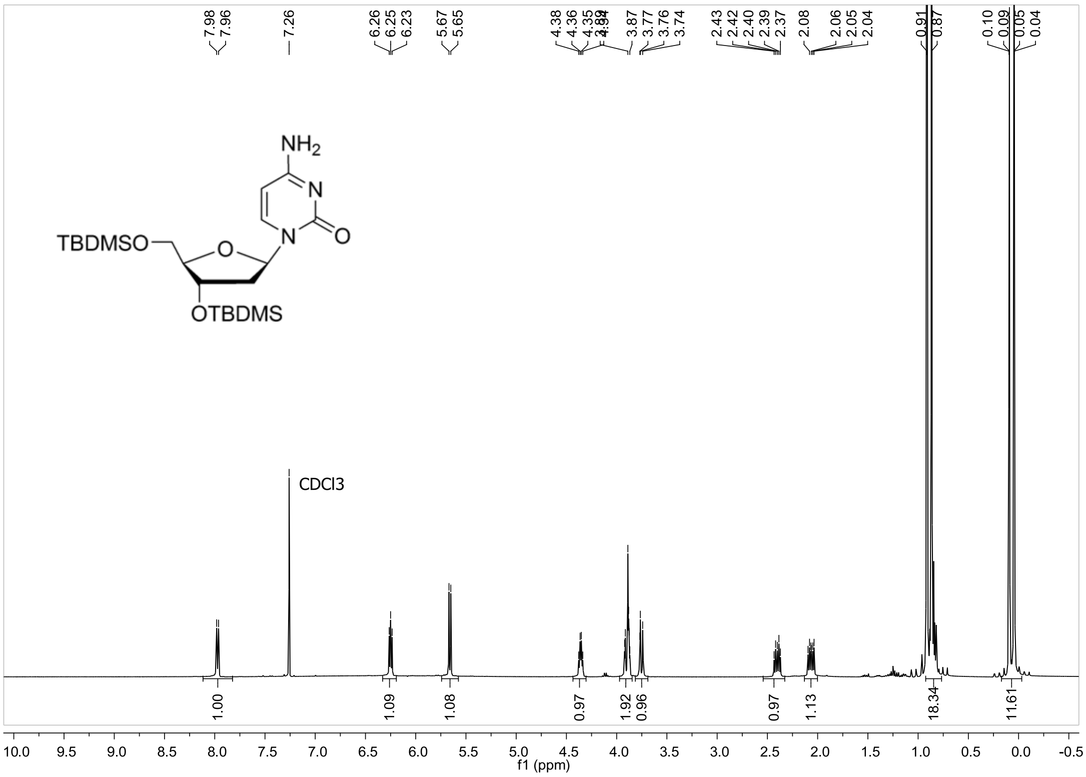

^{13}C NMR spectrum of 11

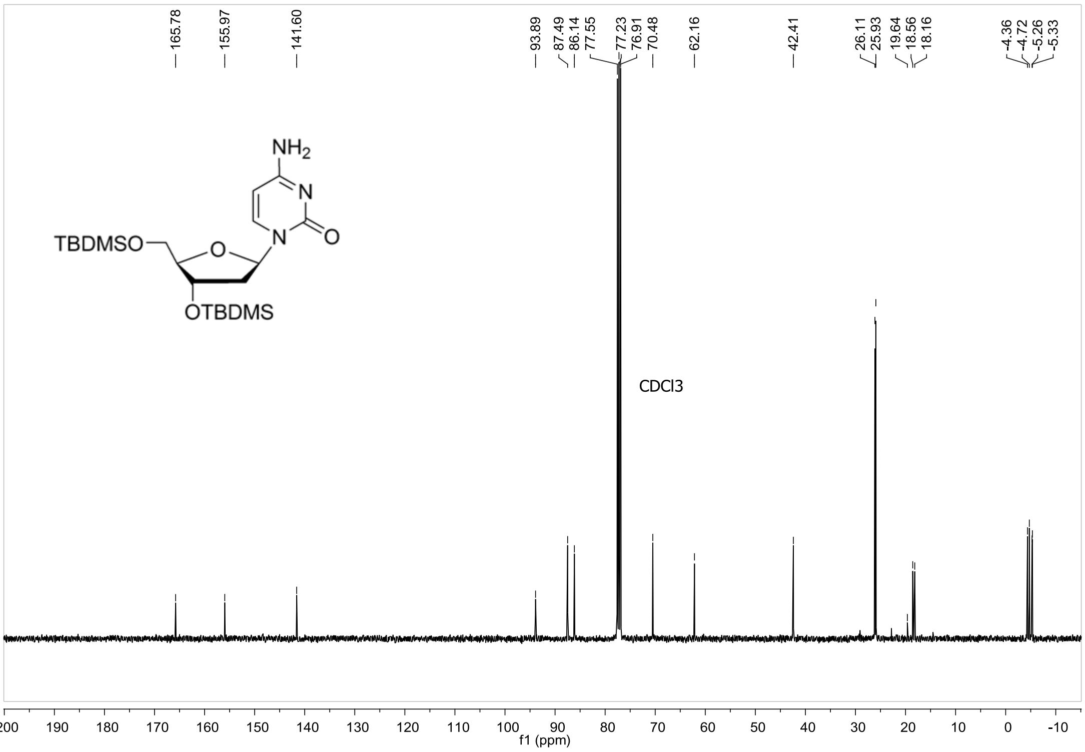

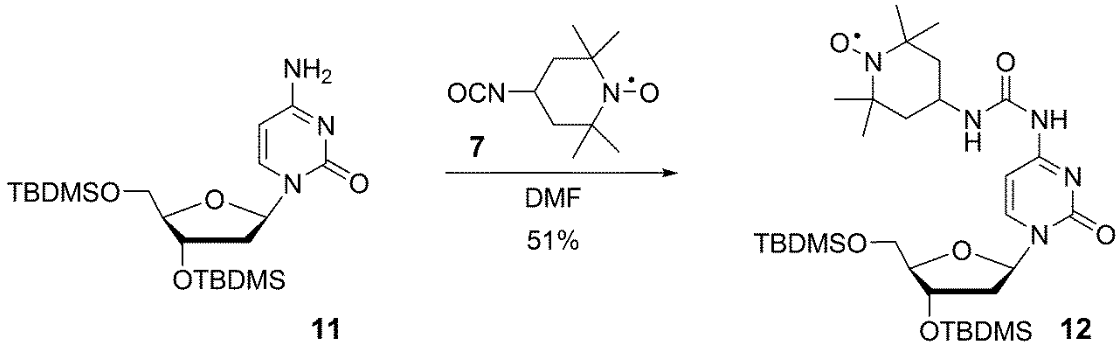

Compound 12. To a solution of compound **11** (555.0 mg, 1.22 mmol) in DMF (2.0 mL) was added 4-isocyanato-TEMPO (160.0 mg, 0.81 mmol) and the resulting mixture was stirred for 9 h at 22 °C. The solvent was removed *in vacuo* and the crude product was purified by flash silica gel column chromatography using a gradient elution (CH_2Cl_2:MeOH; 100:0 to 95:5) to give compound **12** as a light brown solid (270.0 mg, 51% yield).

^{1}H NMR (400 MHz, $CDCl_3$): δ 11.00 (br s), 8.31 (br s), 7.72 (br s), 6.45 (br s), 4.51 (br s), 4.08 (br s), 4.04 (br s), 3.90 (br s), 2.57 (br s), 2.15 (br s), 1.36 (br s), 1.03 (br s), 0.22 (br s).

^{13}C NMR (101 MHz, $CDCl_3$): δ 163.38, 154.70, 153.19, 140.91, 95.43, 86.26, 84.93, 69.13, 60.47, 40.98, 24.26, 16.64, 16.30, -5.37, -6.23, -7.03, -7.18.

HR-ESI-MS $(M + H)^+$: calcd. for $C_{31}H_{59}N_5O_6Si_2$ 653.3998, found 653.4031.

^{1}H NMR spectrum of 12

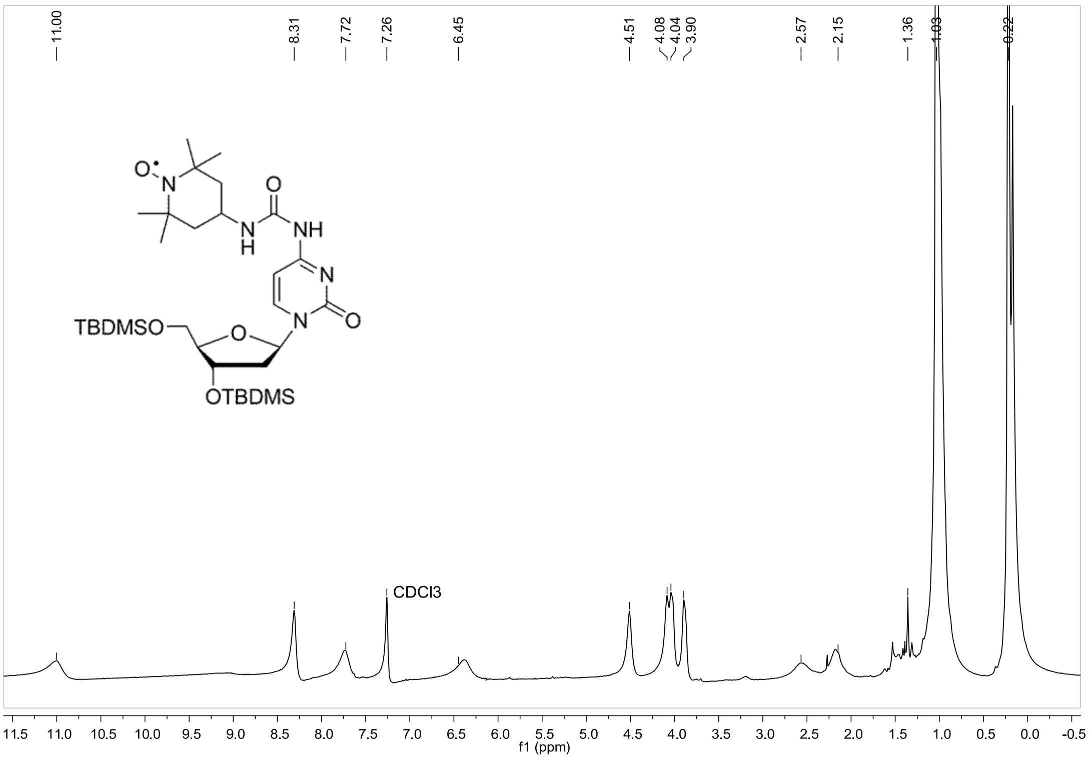

^{13}C NMR spectrum of 12

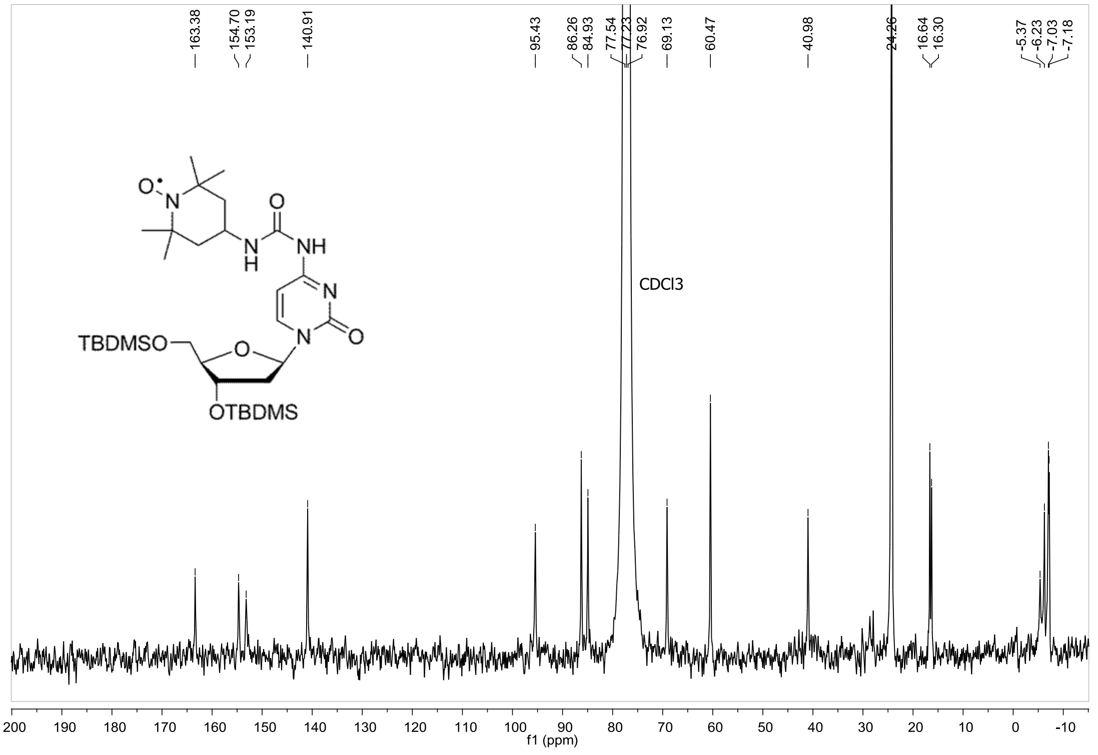

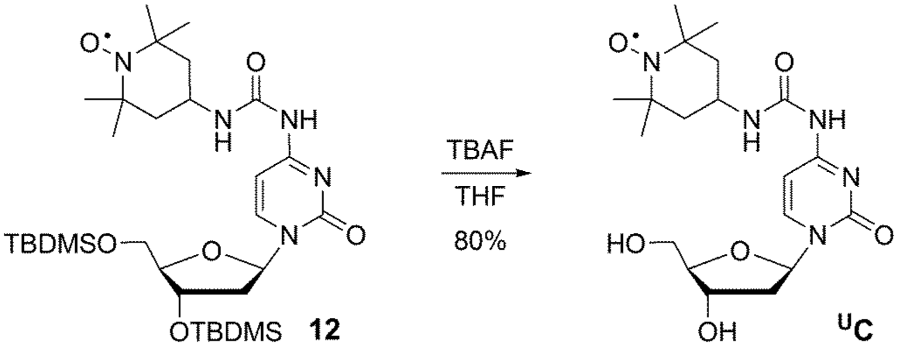

Spin-labeled nucleoside ^{U}***C.*** To a solution of compound **12** (270.0 mg, 0.41 mol) in THF (1 mL) was added tetrabutylammonium fluoride (0.238 mL, 0.87 mol) and the resulting mixture was stirred at 22 °C for 16 h. The solvent was removed *in vacuo* and the residue was purified by flash silica gel column chromatography using a gradient elution (CH_2Cl_2:MeOH; 100:0 to 90:10) to give spin labeled nucleoside $^{\mathbf{U}}$**C** as an orange solid (140.0 mg, 80% yield).

^{1}H NMR (400 MHz, CD_3OD): δ 8.33 (br s), 6.22 (br s), 5.45 (br s), 4.37 (br s), 3.99 (br s), 3.81 (br s), 3.14 (br s), 2.46 (br s), 2.17 (br s), 1.90 (br s), 1.39 (br s), 1.28 (br s), 1.18 (br s), 0.90 (br s), 0.63 (br s).

^{13}C NMR (101 MHz, CD_3OD): δ 164.04, 156.98, 144.95, 97.34, 89.43, 88.48, 71.74, 62.65, 60.25, 59.99, 55.92, 42.56, 32.85, 32.73, 30.98, 23.70, 23.54, 20.36, 14.50, 9.59, 8.60.

HR-ESI-MS $(M + H)^+$: calcd. for $C_{19}H_{31}N_5O_6$ 425.2269, found 425.2243.

^{1}H NMR spectrum of ^{U}C

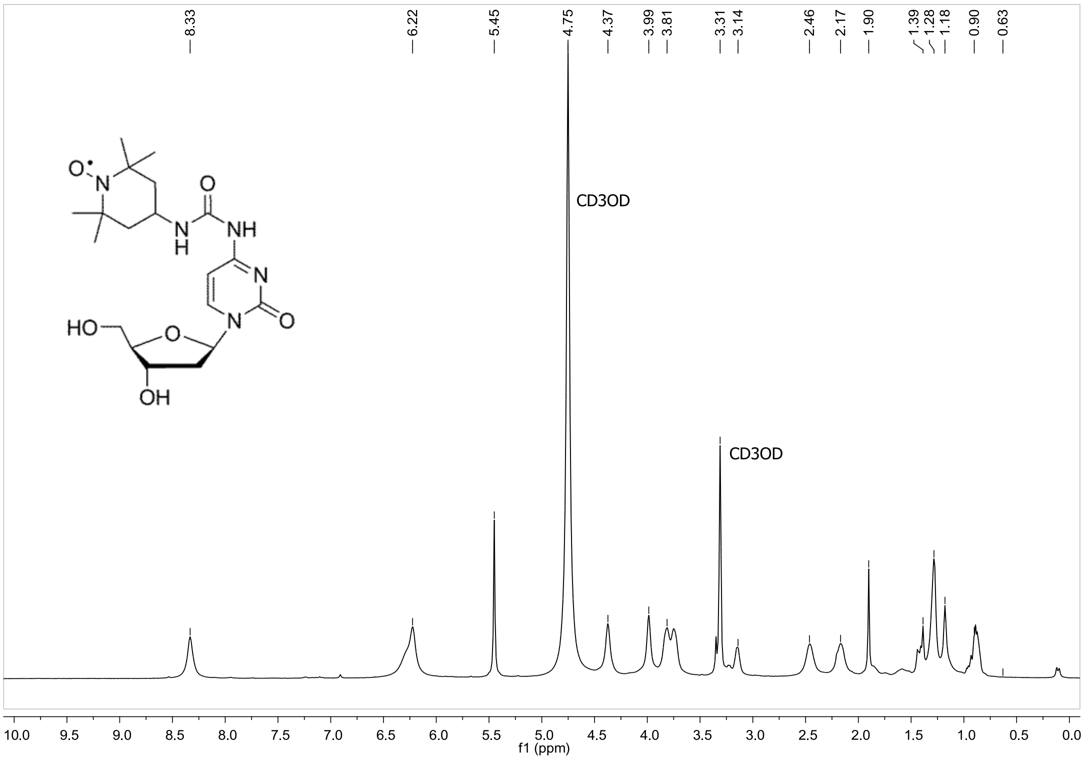

^{13}C NMR spectrum of ^{U}C

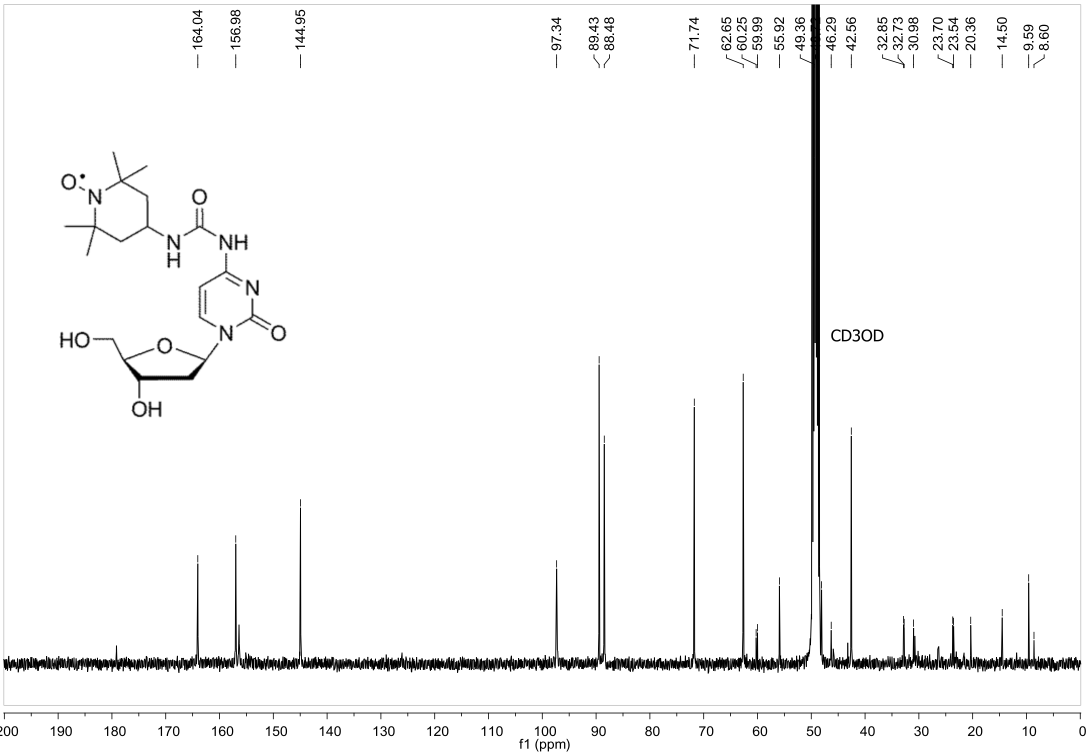

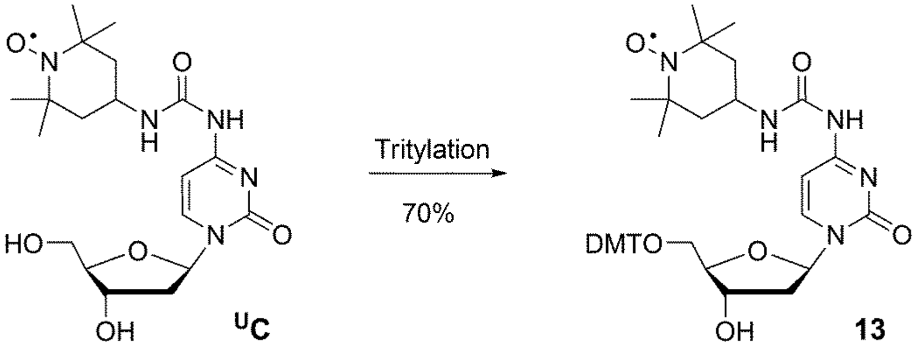

DMT-^{U}C (13). **^{U}C** (100.0 mg, 0.24 mmol), DMTCl (144.0 mg, 0.42 mmol) and 4-DMAP (2.9 mg, 0.024 mmol) were placed in a round bottom flask and kept under vacuum for 20 h. Pyridine (2.5 mL) was added and the resulting solution was stirred at 22 °C for 3 h, after which MeOH (100 μL) was added and the solvent was removed *in vacuo*. The crude compound was purified by flash silica gel column chromatography using a gradient elution of (CH_2Cl_2:MeOH; 100:0 to 97.5:2 + 0.5% Et_3N), using a silica gel column prepared in 99.5% CH_2Cl_2 + 0.5% Et_3N, yielding **DMT-^{U}C** (**13**) as an orange solid (120.0 mg, 70% yield).

^{1}H NMR (400 MHz, $CDCl_3$): δ 10.90 (br s), 8.12 (br s), 7.36 (br s) , 7.26 (br s), 6.81 (br s), 6.41 (br s), 5.22 (br s), 3.76 (br s), 3.37 (br s), 2.94 (br s), 2.49 (br s), 0.98 (br s).

^{13}C NMR (101 MHz, $CDCl_3$): δ 157.40, 153.15, 148.40, 142.80, 141.38, 134.21, 128.81, 126.97, 125.95, 112.18, 105.88, 85.65, 61.58, 54.50, 44.93, 38.51, 10.17.

HR-ESI-MS $(M + H)^{+}$: calcd. for $C_{40}H_{49}N_5O_8$ 727.3576, found 727.3544.

^{1}H NMR spectrum of DMT-[U]C

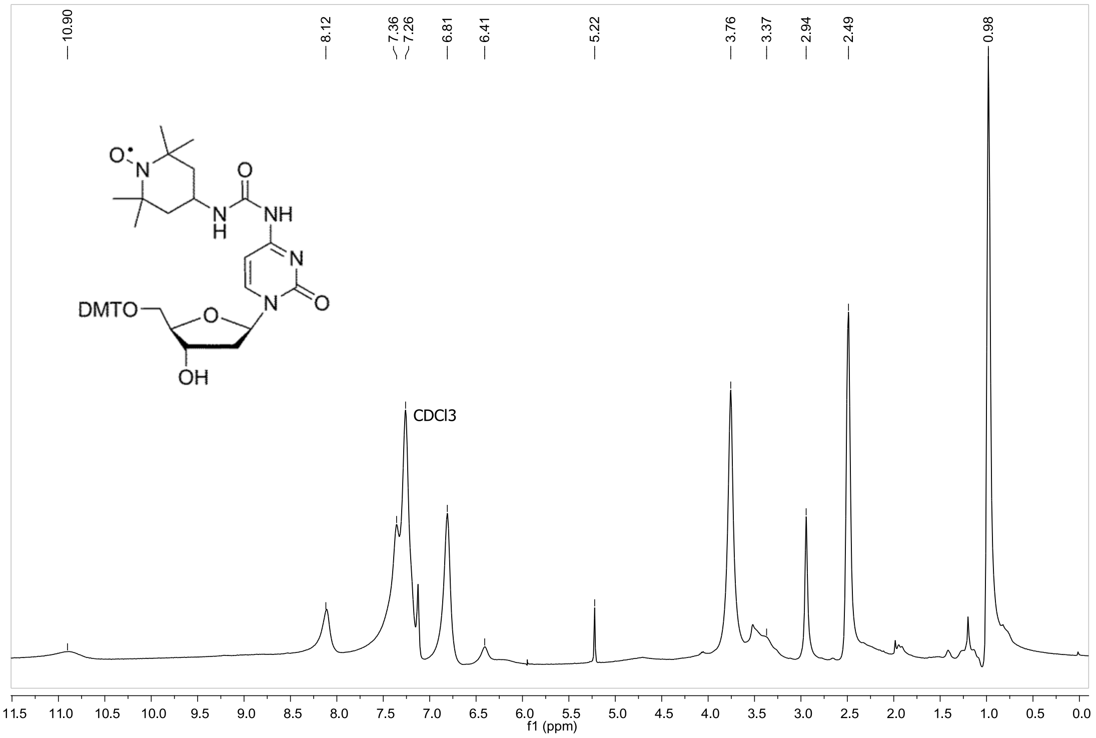

^{13}C NMR spectrum of DMT-[U]C

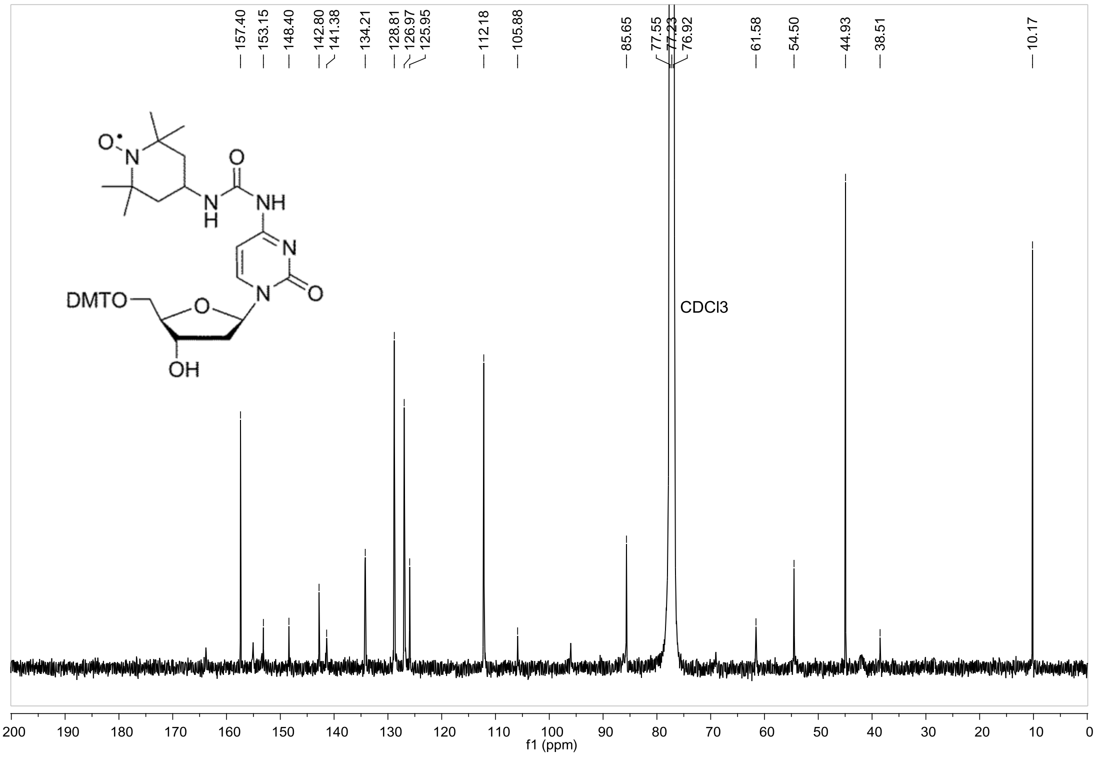

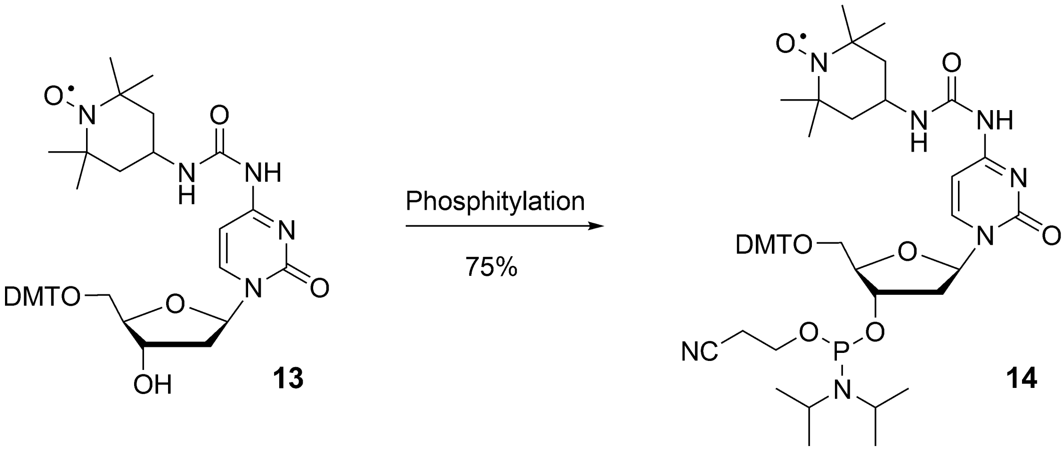

^{U}C phosphoramidite (14). **DMT-^{U}C** (**13**) (70.0 mg, 0.096 mmol) and diisopropylammonium tetrazolide (17.0 mg, 0.13 mmol) were dissolved in pyridine (2 mL) and the pyridine removed *in vacuo*. The residue was kept *in vacuo* for 19 h, followed by dissolution in CH_2Cl_2 (3.5 mL) and addition of 2-cyanoethyl *N*,*N*,*N'*,*N'*-tetraisopropyl phosphoramidite (40.2 mg, 0.13 mmol). The reaction mixture was stirred at 22 °C for 3 h, diluted with CH_2Cl_2 (10 mL) and washed successively with saturated aq. $NaHCO_3$ (3 x 10 mL) and saturated aq. NaCl (3 x 10 mL). The organic solution was dried over anhydrous Na_2SO_4, filtered and concentrated *in vacuo*. The residue was purified by precipitation by first dissolving it in diethyl ether (1 mL) followed by addition of *n*-hexane (80 mL). The liquid was decanted and the operation repeated twice to furnish phosphoramidite **14** as an orange solid (75.0 mg, 75% yield).

^{1}H NMR (400 MHz, $CDCl_3$): δ 10.93 (br s), 8.19 (br s), 7.36 (br s), 7.26 (br s), 6.81 (br s), 6.28 (br s), 4.59 (br s), 4.17 (br s), 3.76 (br s), 3.53 (br s), 3.32 (br s), 2.25 (br s), 1.99 (br s), 1.37 (br s), 1.11 (br s), 0.82 (br s), 0.02 (br s).

^{31}P NMR (162 MHz, $CDCl_3$): δ 150.89, 149.40, 14.59, 8.94.

HR-ESI-MS $(M + H)^+$: calcd. for $C_{49}H_{66}N_7O_9P$ 927.4654, found 927.4621.

^{1}H NMR spectrum of 14

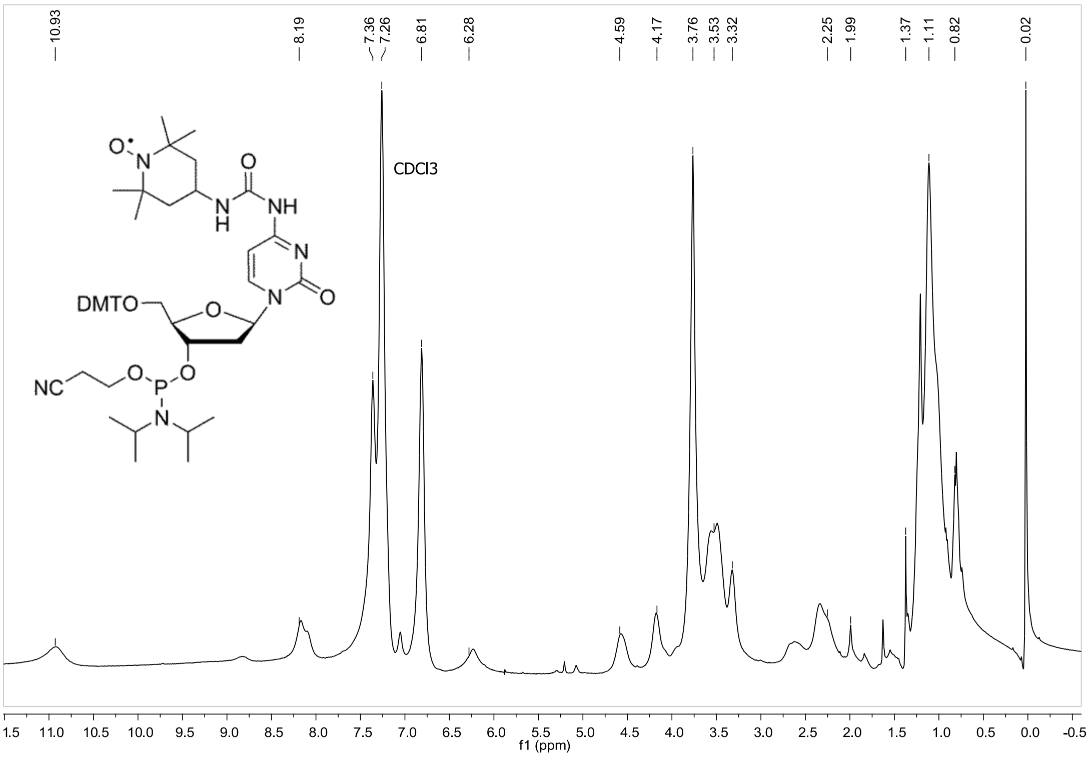

^{31}P NMR spectrum of 14

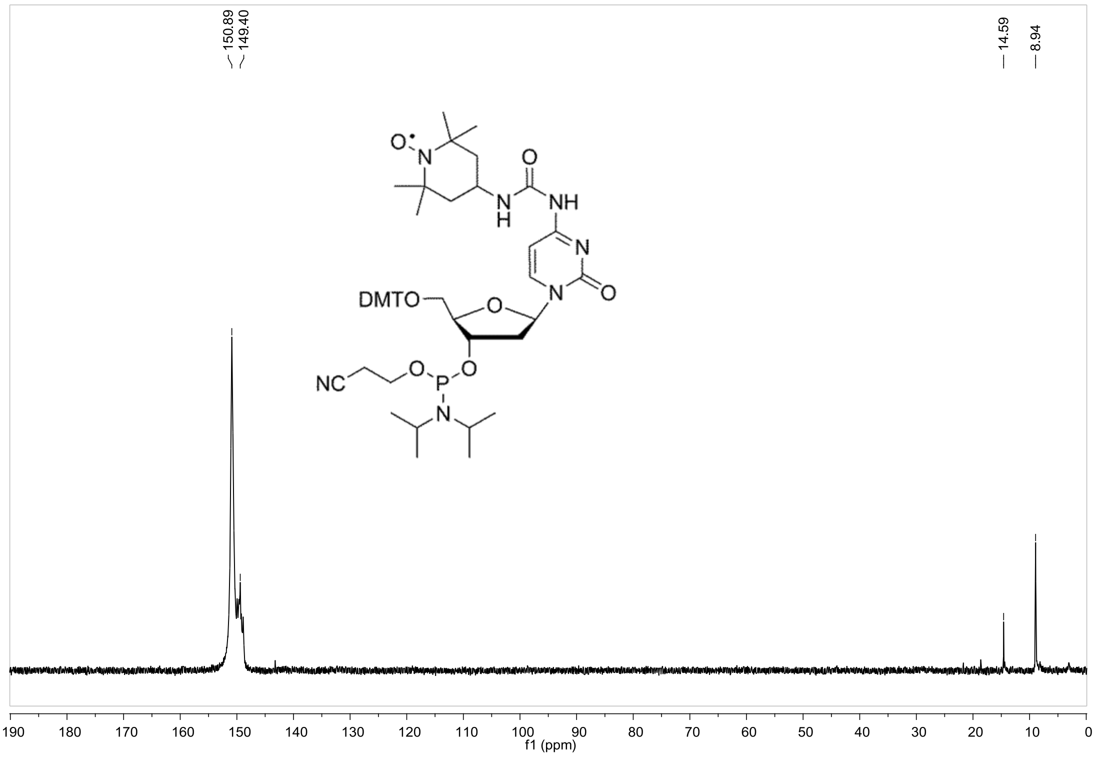

References

1. Ding, P.; Wunnicke, D.; Steinhoff, H.-J.; Seela, F. *Chem. Eur. J.* **2010**, *16*, 14385-14396. doi: 10.1002/chem.201001572
2. Cekan, P.; Sigurdsson, S. T. *J. Am. Chem. Soc.* **2009**, *131*, 18054-18056. doi: 10.1021/ja905623k
3. Jakobsen, U.; Shelke, S. A.; Vogel, S.; Sigurdsson, S. T. *J. Am. Chem. Soc.* **2010**, *132*, 10424-10428. doi: 10.1021/ja102797k
